# Denatonium Benzoate-Induces Oxidative Stress in the Heart and Kidney of Chinese Fast Yellow Chickens by Regulating Apoptosis, Autophagy, Antioxidative Activities and Bitter Taste Receptor Gene Expressions

**DOI:** 10.3390/ani9090701

**Published:** 2019-09-19

**Authors:** Enayatullah Hamdard, Zhicheng Shi, Zengpeng Lv, Ahmadullah Zahir, Quanwei Wei, Mohammad Malyar Rahmani, Fangxiong Shi

**Affiliations:** 1 College of Animal Science and Technology, Nanjing Agricultural University, Nanjing 210095, China; 2017105117@njau.edu.cn (E.H.); 2017105023@njau.edu.cn (Z.S.); lvzengpeng@njau.edu.cn (Z.L.); weiquanwei@njau.edu.cn (Q.W.); 2017107186@njau.edu.cn (M.M.R.); 2 College of Food Science and Technology, Nanjing Agricultural University, Nanjing 210095, China; 2017108149@njau.edu.cn

**Keywords:** denatonium benzoate, bitter taste receptors, apoptosis, autophagy, heart, kidney, chicken

## Abstract

**Simple Summary:**

Denatonium benzoate is a strong bitter taste receptor agonist, extensively used for its activation of different cell pathways. Taste signals have been associated to food recognition and avoidance, and bitter taste provokes an aversive reaction and is assumed to protect chickens from consuming poisons and harmful toxic substances. The results of the study revealed that dietary supplementation with medium and high doses of denatonium benzoate damaged the epithelial cells of the heart and kidneys by inducing apoptosis and autophagy and reduced the growth of chickens, respectively. However, mRNA expressions of bitter taste receptors, downstream signaling effector genes, apoptosis-, autophagy- and antioxidant-related genes were higher on day 7, while these expressions were subsequently decreased on day-28 in the heart and kidney of Chinese Fast Yellow chickens in a dose-response manner.

**Abstract:**

The sense of taste which tells us which prospective foods are nutritious, poisonous and harmful is essential for the life of the organisms. Denatonium benzoate (DB) is a bitter taste agonist known for its activation of bitter taste receptors in different cells. The aim of the current study was to investigate the mRNA expressions of bitter taste, downstream signaling effectors, apoptosis-, autophagy- and antioxidant-related genes and effector signaling pathways in the heart/kidney of chickens after DB dietary exposure. We randomly assigned 240, 1-day-old Chinese Fast Yellow chicks into four groups with five replicates of 12 chicks and studied them for 28 consecutive days. The dietary treatments consisted of basal diet and feed containing DB (5, 20 and 100 mg/kg). The results revealed that dietary DB impaired (*p* < 0.05) the growth performance of the chickens. Haemotoxylin and eosin staining and TUNEL assays confirmed that medium and high doses of DB damaged the epithelial cells of heart/kidney and induced apoptosis and autophagy. Remarkably, the results of RT-PCR and qRT-PCR indicated that different doses of DB gradually increased (*p* < 0.05) mRNA expressions of bitter taste, signaling effectors, apoptosis-, autophagy- and antioxidant- related genes on day 7 in a dose-response manner, while, these expressions were decreased (*p* < 0.05) subsequently by day-28 but exceptional higher (*P* < 0.05) expressions were observed in the high-dose DB groups of chickens. In conclusion, DB exerts adverse effects on the heart/kidney of chickens in a dose-response manner via damaging the epithelium of the heart/kidney by inducing apoptosis, autophagy associated with bitter taste and effector gene expressions. Correlation analyses for apoptosis/autophagy showed agonistic relationships. Our data provide a novel perspective for understanding the interaction of bitter taste, apoptosis, autophagy and antioxidative genes with bitter taste strong activators in the heart/kidney of chicken. These insights might help the feed industries and pave the way toward innovative directions in chicken husbandry.

## 1. Introduction

Taste is well-known biological descriptor for sweet, bitter, sour, salty receptors and ion channels, which plays a critical role in the life and nutritional status of chickens and other organisms. Bitter taste perception provides animals with critical protection against the ingestion of poisonous and harmful toxic compounds [1]. Taste signals have been associated to food avoidance and recognition, as well as feed or liquid intake in different species of animals [2,3,4]. With the simultaneous inflation in the cost of animal feed and higher standards of livestock products, people endeavor to discover novel feed additives and effective alternatives to traditional antibiotics [5,6]. Efforts have been made to extract incredible numbers of potential additives from natural plants, and they often display bitter taste. However, bitter taste receptors are part of a superfamily which includes more than twenty members. This makes very difficult to do functional studies for each bitter taste receptor. In addition, bitter taste receptors expressions are not limited to taste buds but also exist in extra-gustatory organs, so it is critical to determine their extra-gustatory functions. Several studies have already revealed that bitter taste receptors exert a variety of functions in different cells and tissues [7,8]. Chickens have only three bitter taste receptors: ggTas2r1, ggTas2r2 and ggTas2r7 [9]. Due to their low number of bitter taste receptors, chickens are a good minimalistic model for understanding the functions of bitter taste receptors in non-gustatory tissues [9]. Denatonium benzoate as a bitter taste receptor stimulus can bind to the bitter taste receptors to activate bitter taste signaling. Furthermore, it has been reported that this receptor family plays a critical role in the heart [10], thyroid [11], and gastrointestinal muscle [12]. However, their roles in the kidney has never been determined. Therefore, our present project aimed to examine the roles and underlying mechanisms of bitter taste transduction signaling associated with mRNA expression patterns of bitter taste receptors, apoptosis-, autophagy- and antioxidant-related genes in the hearts and kidneys of chickens.

Cysteine aspartate-specific proteases (CASPs or caspases) serve as intrinsic initiators of apoptosis by cleaving substrates at aspartate residues [13]. In mammals and humans the caspase protein family currently consists of 13 and 11 isoenzymes, respectively [14]. The function of caspases is closely associated to the initiation and execution of apoptosis, with caspases categorized as either initiator or effector caspases. CASP2, CASP8, CASP9 and CASP10 are initiator caspases, and CASP3, CASP6 and CASP7 are effector caspases [15]. Known as interleukin 1β-converting enzyme, CASP1 plays an important role in both apoptosis and inflammation [16]. Apoptosis is regulated by stepwise activation of caspases for the processing or cleaving of other caspases [17].

Programmed cell death or apoptosis has a vital role in various biological events. This is a process of single cell death controlled by the activation of specific genes, the elimination of unwanted or damaged cells by apoptosis is an indispensable action that occurs via several mechanisms which maintain cellular homeostasis, and normal regulation of the immune system [18]. An earlier study showed that denatonium benzoate enhanced intracellular Ca^2+^, damaged mitochondria and induced apoptosis in airway epithelial cells, respectively [19]. Programmed cell death is activated by intracellular stresses and developmental cues. Well-known representative intrinsic regulators, the extended BCL2 family proteins, play crucial roles in cell death regulation and are able to regulate several cell death mechanisms, including apoptosis, necrosis and autophagy [20,21,22]. Caspase-independent mechanisms lead to the release of apoptosis-inducing factor (AIF) from the mitochondria, inducing large-scale DNA fragmentation in several cell types including heart and kidney cells to induce apoptosis caused by AIF [22]. BCL2 family proteins contain at least one of four BCL2 homology (BH) domains (i.e., BH1, BH2, BH3 or BH4), and the number of BH domains included in proteins is associated with their apoptotic functions [23].

Autophagy is the pathway involved in forming an organelle called an autophagosome. This pathway moves something from the cytoplasm of the cell into the lysosome for degradation. The term, which derives from the Greek words ‘auto’ meaning self and ‘phagos’ meaning to eat, is defined as a catabolic pathway involving the degradation of cellular components via the lysosomal machinery [24,25]. Autophagy is the natural process by which the cells can clear out damaged mitochondria, recycle proteins, and get rid of intracellular pathogens. However, all organisms need a balance of autophagy with anabolic processes. There are about 30+ different proteins involved in the formation of the autophagosome. Researchers are still actively figuring out how all of the bits and pieces of the process go together, but recent genetic studies have shed a lot of light on the pathway. A family of genes known as the autophagy-related genes, whose abbreviations start with A/TG, code for several of the proteins integral to autophagy. Several of these genes have variants that have been studied in reference to pathogen susceptibility, autoimmune diseases, cancer, and sepsis [26,27,28].

There is a complex system containing natural enzymatic and non-enzymatic antioxidants that protect the body from oxidative damage. Briefly, the antioxidant enzymes (SOD, MnSOD, CAT, GSH, and GSH-Px) appear to be the first line of defense during oxidative stress, and exert beneficial effects preventing oxidative damage in poultry raising [29,30,31]. Catalase (CAT) participates in defense mechanisms against oxidative stress by converting H_2_O_2_ into water and molecular oxygen [32]. The antioxidant glutathione peroxidase enzymes (GPX) are implicated in the protection of cells against oxidative damage by reducing H_2_O_2_ and other organic peroxidases to water with reduced glutathione [32].

Denatonium benzoate (DB), one of the most bitter-tasting substances known, is described as extremely unpleasant at a lower amount and can cause perceptible bitterness [33]. DB has been demonstrated extensively as a bitter taste agonist and used to activate bitter taste receptors in many cell types, including taste cells [34]. DB is intensely bitter and non-toxic, and can be detected by human taste receptors [35]. For these reasons DB has been broadly added to liquid detergents, cosmetics, plastic toys and personal care products to avoid the consumption of harmful substances [36]. Aside from its strong bitter taste, DB also exerts biologic effects on various physiological systems in different organisms. Exposure to DB quickly suppressed ongoing intake and delayed gastric emptying in rodents [37,38].

However, there is limited knowledge about the biologic effects of DB and its potential molecular mechanisms in chicken heart and kidneys and no studies have been conducted to date to investigate the relationship between bitter receptors, signaling effectors, and apoptosis-, autophagy- and antioxidant- related genes in the chicken heart and kidney. Therefore, the objective of this study was to investigate the mRNA expressions of bitter taste and its downstream signaling effectors, apoptosis-, autophagy-, and antioxidant-related genes and transduction signaling pathways in chicken heart and kidney to DB dietary exposure in a dose-response manner. The results were confirmed by RT-PCR, qRT-PCR, haemotoxylin and eosin, TUNEL assay, correlation of apoptosis, autophagy and bitter taste receptors and its associated downstream signaling pathway, complete amino acid sequence alignments of related genes, selected gene heat mapping and the potential molecular mechanisms of action of dietary exposure to denatonium benzoate on the heart and kidney cells of Chinese Fast Yellow chickens were determined.

## 2. Materials and Methods

### 2.1. Chemicals

Denatonium benzoate (98%) was purchased from Adamas Reagent Co. Ltd. (Nanjing, China) and then stored at room temperature until the end of the experiments. RNA0se (phenol 38%), isoprophyl alcohol, trichloromethane, 100% absolute alcohol, DNA/RNAase-free water all were purchased collectively from RNA detection from (Takara Bio Inc., Shiga, Japan), Cat. # RR047A v 201810Da PrimeScript™ RT reagent Kit with gDNA Eraser (Perfect Real Time) and Cat. # RR420A v201810Da, TB Green™ Premix Ex Taq ™ (Tli RNase H Plus), were both purchased from TaKaRa (Dalian, China). 

### 2.2. Birds Management and Treatments

A total of 260 1-day-old local Chinese Fast Yellow chicks were housed individually in the Animal Room of Nanjing Agricultural University under standard conditions, and were randomly divided into four (4) treatment groups: Control group (Control), low-dose DB-treated group (5 mg/kg, Low-Den), Medium-dose DB-treated group (20 mg/kg, Medium-Den) and high-dose DB-treated group (100 mg/kg, High-Den), respectively. The control group was fed with basic corn-soybean diet according to NRC (1994) (Table 1). The Low-Den, Medium-Den and High-Den treatment groups were fed the same commercial diet with denatonium added at 5, 20 and 100 mg/kg, respectively. Each treatment includes five cages, and each cage consisted of 12 chicks. Chicks were reared in a ventilated chicken house in which the lighting regime was 16-h light:8-h dark, the relative humidity was approximately 50 ± 5% and chickens were offered formulated feed (Table 1) ad libitum with freely available tap water for 28 consecutive days. Average body weight was calculated on day 1, day 07, day 14 and day 28, respectively. The experimental protocol and procedures were designed and approved in accordance with the Guidelines for the Care and Use of Animals prepared by the Institutional Animal Care and Ethical Committee for Nanjing Agricultural University (Permit number: SYXK (Su) 2019-0047), Nanjing, China.

### 2.3. Feed Mixing and Formulation

A basic corn-soybean diet with the ingredients listed in Table 1 was purchased from ADM (Nanjing, China). 

The feed was mixed with a manual electric mixing machine available in the Nanjing Agricultural University animal house, according the experimental design. 

### 2.4. Sample Collection

On days 7 and 28 of age (starter and grower stages), 10 chickens with body weights near the average of their group were slaughtered via exsanguination. The heart and kidneys were gingerly separated, and immediately cut up into two sections. One section was promptly fixed in 4% paraformaldehyde for histological analyses, whereas the second section was stored at −80 °C for the analysis of gene expression.

### 2.5. TUNEL Assay

The terminal deoxynucleotidyl transferase-mediated deoxy uridine triphosphate nick-end labeling (TUNEL) assay was carried out referring to the kit manufacturer’s instructions. Heart and kidney apoptosis were determined using a TUNEL Bright Green Apoptosis Detection Kit (A112, Vazyme Biotech, Nanjing, China). According to the protocol, the paraffin sections of heart and kidney were deparaffinized, rehydrated and then incubated with Proteinase K (20) at room temperature for 20 min. Later, the sections were incubated with TdT enzyme buffer containing double distilled H_2_O, eecombinant TdT enzyme, equilibration buffer and Bright Green Labeling Mix at 37 °C for 60 min under dark conditions. Finally, after washing three times in PBS, the sections were stained with 4′,6-diamidino-2-phenylindole (DAPI) staining solution (C1005, Beyotime Biotechnology, Shanghai, China) for 5 min under dark conditions. The negative control was prepared as above without incubation of the TdT enzyme buffer to ensure no non-specific reaction occurred during the experiments. Images were taken through a LSM 700 confocal laser scanning microscope (Carl Zeiss, Oberkochen, Germany). The total numbers of apoptotic cells (green color) and total cells (blue color) were counted using the Image-Pro Plus software 6.0. The apoptotic index was defined as the ratio of apoptotic cells to total cells.

### 2.6. Body and Organ Weight Measurements

**Figure 1:**
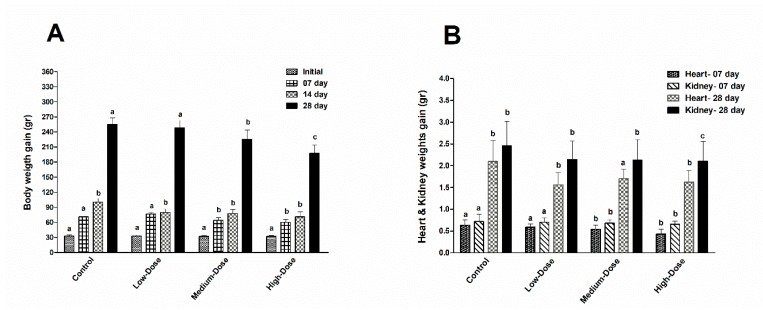
Effects of DB supplementation on the body and organ weight gain (gr) of chickens. (**A**) Body weight of chickens at 0, 07, 14, 28 days of age (n = 10). (**B**) Organ weights of chickens at day 7 and day 28 of age (n = 10). Data are presented as mean value ± SEM. Values without the same mark (a, b, c) represent statistically significant differences (*p* < 0.05). Subfigure A uses line graphs with experimental days on the *X*-axis for body weights, and subfigure B separates the data into two bar graphs for heart and kidney, respectively.

All chicken live body weights were measured at four different stages on days, 1, 7, 14 and 28 of the experiment and a total 10 chicken in each group (two chicken/replicate) were used in each stage of killing to collect and measure heart and kidney weights at day 07 (starter stage) and at day 28 (grower stage), respectively (Figure 1).

### 2.7. Haemotoxylin and in Eosin Staining (Histological Observations)

To observe histological changes, heart and kidney samples were fixed in 4% paraformaldehyde for 12 h, then dehydrated through a graded ethanol series, cleared with xylene, embedded in paraffin wax, and serially sectioned at 4-μm thickness. The sections were stained with H&E, and histopathological changes were then viewed under a YS100 microscope (Nikon, Tokyo, Japan). Four (4) sections from each stage were observed for determination of apoptotic and autophagic cells in the tissues as reported previously [39].

### 2.8. Total RNA Extraction and mRNA Quantification

The total RNA from heart and kidney for RT-PCR and real-time PCR was extracted and purified from frozen collected tissues using RNAose (Takara Bio Inc,), which includes gDNA Eraser (Perfect Real Time) for elimination of genomic (g) DNA according to the manufacturer’s protocols. The concentration and quality of total RNA was identified by a micro-spectrophotometer (Thermo Scientific, Wilmington, DE, USA). The samples with the 260/280 ratios of 1.8–2.0 and 260/230 ratios of 2.0–2.2 were chosen for further RT and qRT-PCR reactions. Afterward, mRNA was reverse transcribed into complementary DNA (cDNA) using the PrimeScript™ RT reagent Kit with gDNA Eraser (Perfect Real Time, Cat. # RR047A v201810Da) in accordance to the manufacturer’s instructions. Gene-specific primers for bitter taste receptors (ggTas2Rs), apoptosis related genes, autophagy genes and antioxidant genes were generated with aid of the nucleotide database of The National Center for Biotechnology Information (NCBI) [40]. according to their published cDNA sequences (Table 2). The target genes and the housekeeping gene were synthesized by Sangon (city, country) and applied for real-time PCR and their primer sequence is shown in Table 2. Amplicon lengths for real-time PCR were between 92 and 200 bp. The PCR final reaction mixture of 20 μL included 10 μL of TB Green Premix Ex Taq (Tli RNase H Plus) (2×), 0.4 μL of ROX Reference Dye 1 or Dye 2 (50×), 2 μL of cDNA template diluted in ratio of 1:3, 0.4 μL of each primer (10 μM) and 6.8 μL of double distilled H2O (TaKaRa). PCR reactions were performed in 96 well reaction plates on a 7500 Real-time PCR instrument (Applied Biosystems, ABI, Beverly, MA, USA), all genes were assayed three times. under the following conditions: hold stage (95 °C for 30 s), PCR stage (40 cycles of 95 °C/2 min, 60 °C/34 s) apparently, to verify the amplification of a single product, a stage with a temperature increment (Melt Curve Stage) was conducted to generate a melting curve under the following conditions: (95 °C/15 s, 60 °C/1 min), followed by a temperature increment of 95 °C/15 s. Relative gene expression levels were analyzed by the 2***** method after normalization against β-actin.

### 2.9. Statistical Analysis

#### Body and Organs Weights Measurements

Body weight and two (2) selected organs weight measurements were described previously [41]. Significant differences between treatment groups and control group were analyzed by one-way analysis of variance (ANOVA) followed by a Duncan post hoc test. A *p* < 0.05 value was considered as statistically significant, marked with (a–d). Value =MEAN ± SEM, weight unit (g).

### 2.10. Gene Expression Profile

For relative gene expression analysis apoptosis, autophagy, antioxidant, downstream signaling effectors and bitter taste receptor genes for each organ at different growing stages were compared to he chosen control gene (β-actin) in two tissues (heart and kidney) using one-way ANOVA. In addition, multiple comparison among means of mentioned target genes and β-actin gene levels in each group were calculated using Dunnett’s test (marked with a–e) as shown in the respective figures. An alpha level of 0.05 was set for all tests. Results were described as the mean ± standard error of the mean (SEM). *p* < 0.05 was considered to be statistically significant. These statistical analyses were conducted with GraphPad Prism 6 (IBMP Crop, Unites States), and IBM SPSS Statistics, version 20 software (SPSS Inc., Chicago, IL, USA).

## 3. Results

### 3.1. Body and Organ Weights Measurements

After continuous feeding for 28 consecutive days with DB-containing diets or control, the body weights of Chinese Fast Yellow chickens were significantly decreased in the medium-dose (20 mg/kg) and high-dose (100 mg/kg) groups (*p* < 0.05), however, no differences were observed between control and the low-dose (5 mg/kg) groups (Figure 1). After 28 days of dietary exposure, all DB-treated groups showed significantly reduced body weights (*p* < 0.05; Figure 1A,B). However, the average body weight of control and low-dose treated groups were almost similar and no major changes were observed during the experiment. The heart and kidney weight gains were recorded twice during the time of experiment, the heart weight gains on day 07 in the control, low-dose and medium-dose of DB-treated groups were significantly (*p* < 0.05) similar, but reduced in the high-dose DB group, respectively, whereas, the heart weight gain on day-28 in all DB-treated groups were significantly (*P* < 0.05) reduced compared to the control group. In addition, the kidney weight gain on day-07 was similar in all DB-treated groups, interestingly the kidney weight gaining in comparison to heart weight gaining was higher in all groups. Moreover, the kidney weight gain on day-28 was equally reduced in all three DB-treated groups (low-dose, medium-dose and high-dose) but significantly (*p* < 0.05) increased in the control group, respectively (Figure 1A,B).

### 3.2. Histological Observations

#### 3.2.1. Histological Changes in the Heart of Chicken

**Figure 2:**
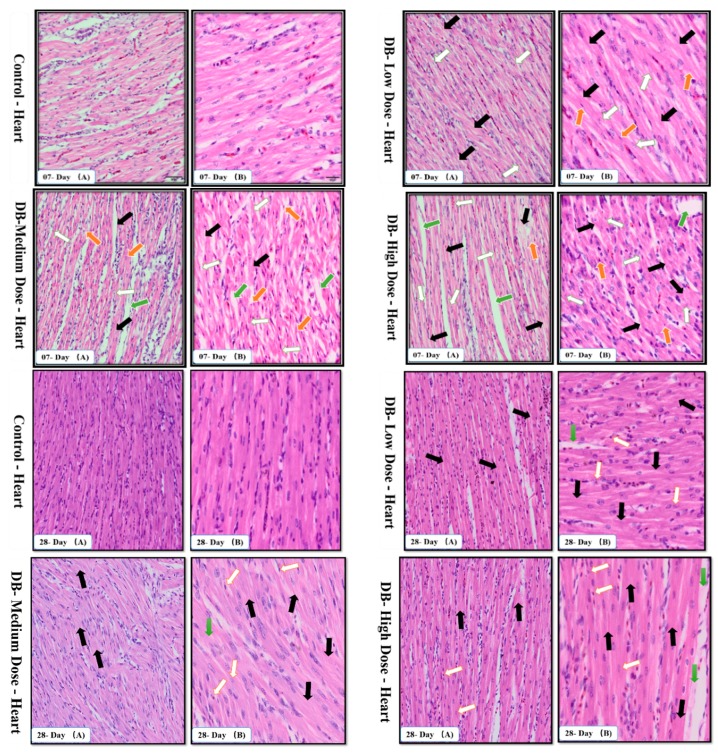
Effects of Denatonium benzoate supplementation on chicken heart histomorphology on days 07 and 28 (**A**,**B**). On day-07 necrosis (red arrow), apoptotic cells (white arrow), pyknotic cells (yellow arrow) and distortion of the morphological characteristics of the cells (green arrow) due to the toxic effect of DB were seen. On day-28, karyolysis due to severe necrosis which caused autophagy (black arrow), apoptotic cells (white arrow) shrinkage of fibroblasts which led to condensation (green arrow) indicate the effect of DB.

To evaluate whether dietary DB exposure caused pathological changes in the hearts of chickens, we examined the heart tissue cellular morphology on days 07 and 28 via H&E staining and observed apoptotic changes. Compared to the control group, there was moderate necrosis, myocardial inflammatory infiltration and pyknotic cells, but no morphological distortion of cardiac cells in the low-dose of DB group (Figure 2A,B). In the medium-dose DB groups, there were greater changes compared to the low-dose ones, while severe changes were observed in the high DB dose groups. We also observed cell necrosis, apoptotic cells (cell damage/death), pyknotic cells, and distortion of the normal morphological characteristics of the cells due to the toxic effects of DB for all respective doses. When the DB dietary treatment was continued for 28 consecutive days these effects were increased, with karyorrhexis and karyolysis due to severe necrosis which caused autophagy, shrinkage of fibroblasts leading to condensation, and the number of apoptotic cells in the hearts of chickens was increased (Figure 2A,B). 

The results indicate that a high dose of DB can cause severe necrosis of the cells, shrinkage, distortion and changes in the characteristics of the cells which finally lead to apoptosis and autophagy, respectively.

#### 3.2.2. Histological Changes in the Kidney of Chicken

**Figure 3:**
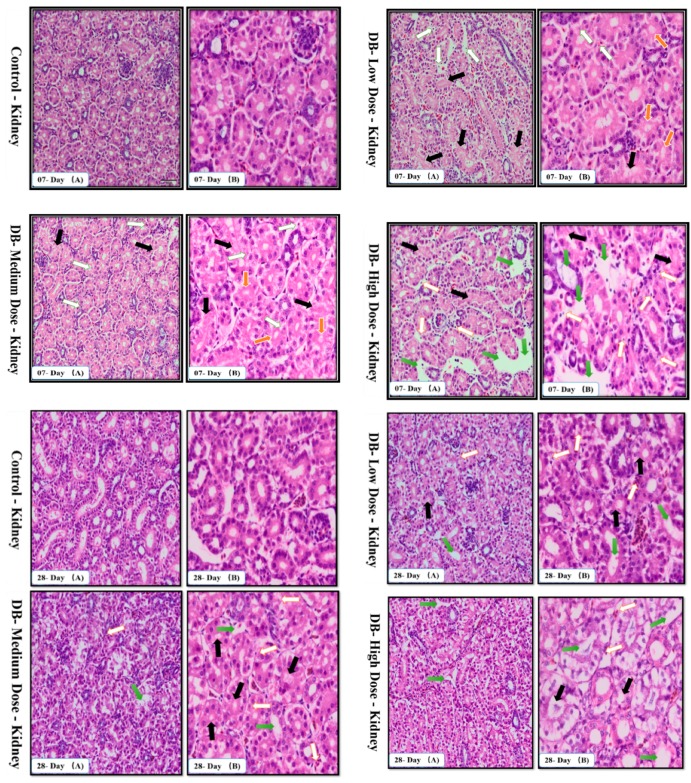
Effects of DB supplementation on the histomorphology of chicken kidney on day-07 and day-28 (**A**,**B**). On day-07, the black arrow shows that PCTs became swollen (hydrophiid degeneration). The white arrow indicates inflamed infiltrated cells and the green arrow indicates separation of the basement membrane of DCTs; Karyolysis occurred where no cells were found due to the toxic effects of DB. The effects on day 28 showed no major differences.

Histological changes are a direct indication of kidney damage. In our study, microstructure studies were performed on chicken kidneys. The chicken kidney microstructure is shown in Figure 3A,B.

By comparing the control group results with the groups treated with three different doses of DB, we found several histological changes in the kidney of chickens on days 07 and 28 (Figure 3A,B). Major changes were observed in the high-dose DB group on day-07 of dietary exposure to DB. These changes included Proximal Convoluted Tubules (PCTs) with swelling due to hydrophiid degeneration and influencing the smaller in size collecting tubes. Inflammed infiltrated cells were also observed and separation of basement membrane of Distal Convoluted Tubules (DCTs) which led to karyolysis, apoptosis and autophagy. No live cells were found in the damamged areas of kidneys due to the toxic effects of medium and high doses of DB (Figure 3A,B). In addition, the chickens were more sensitive during the initial days of DB dietary exposure, therefore the major histological effects were visible on day-07 of exposure rather than day-28 and there were no major differences in terms of histopathological observations on day-07 and day-28 (Figure 3A,B). The results indicated that the chicken kidney is a vital organ for the filtration and excretion of hydrophilic substances inside the body. Therefore, swelling of nephrotic cells were observed which can lead to nephrosis and dysfunction of nephrons, the glomerulus is sensitive to toxic compounds and this can easily lead to inflammation and damage of nephrotic cells.

### 3.3. Confirmation of Apoptosis in the Heart and Kidney of Chicken by TUNEL Assay

**Figure 4:**
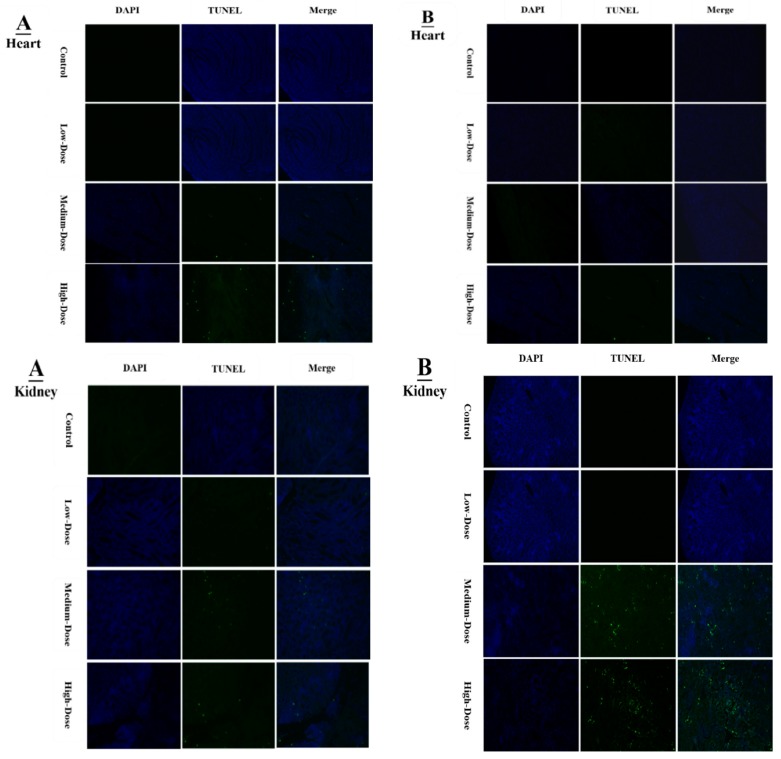
Tunnel assay of heart and kidneys at 07 days (**A**) and at 28 days (**B**) of age by immunofluorescence. The blue color represents the total cells in the heart and kidneys, and the green color represents the apoptosis cells in the heart and kidneys with three different doses of DB (low-dose, medium-dose and high-dose).

The results after staining samples with a TUNEL assay kit are shown in Figure 4A, BTUNEL-positive cells with green colored nuclei represent apoptotic cells.

These apoptotic cells were observed on day-07 and day-28 in the hearts and kidneys of chicken. However, the numbers of apoptotic cells in both hearts and kidneys were higher on both day-07 and day-28 in the medium- and high-dose DB-treatment groups compared to control group (*p* < 0.05; Figure 4A,B). In comparison with the medium-dose group, the high-dose group exhibited a greater percentage of apoptotic cells on day-28 in both hearts and kidneys of the chickens (*p* < 0.05). No significant pathological differences were observed in apoptotic cells in the low-dose group in contrast with the control group on days 07 and 28, respectively.

### 3.4. Determination of Real Time (RT) and Quantitative Real-Time (qRT- PCR) Expressions

#### 3.4.1. The mRNA Expressions of Bitter Taste and Downstream Signaling Effectors in the Heart

**Figure 5:**
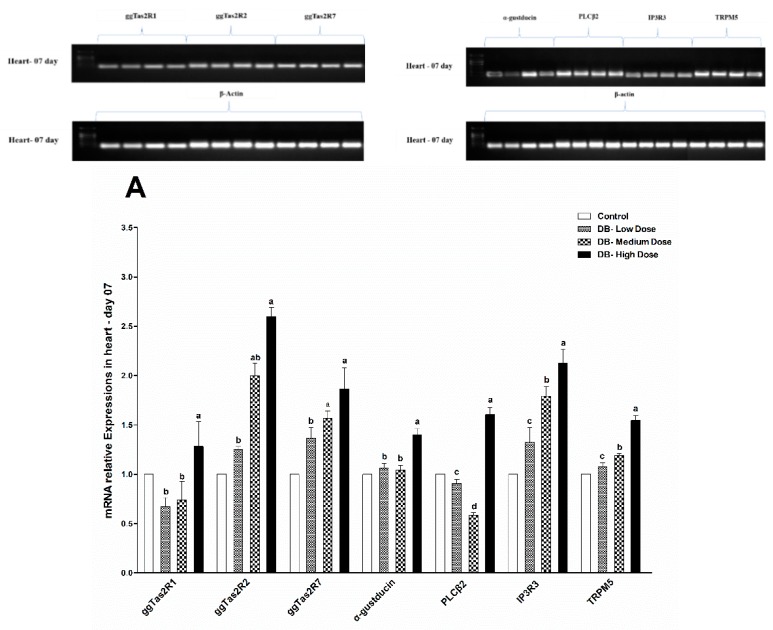

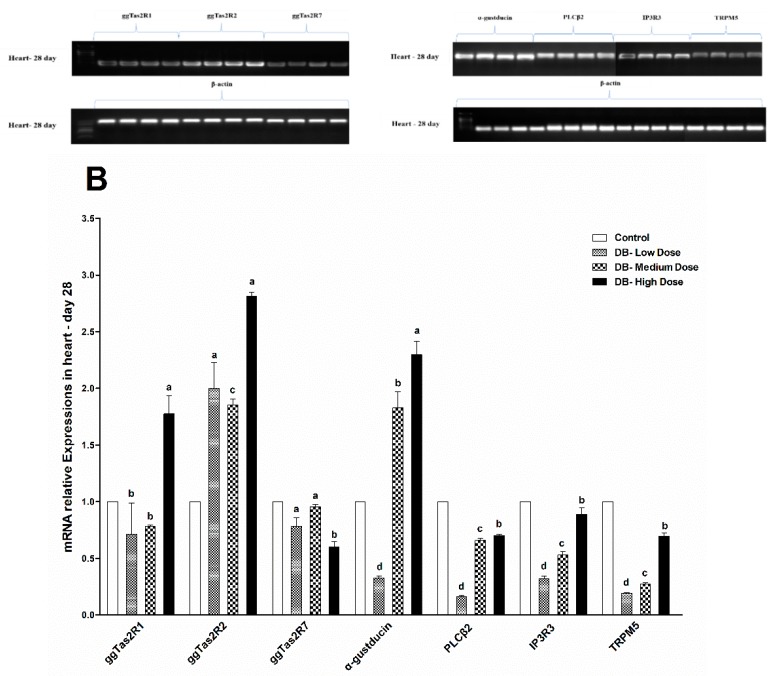
RT and qRT-PCR showed effects of DB supplementation on heart mRNA expressions of bitter taste receptors and downstream effectors at 07 days (**A**) and 28 days (**B**) of age. Data are presented as mean value ± SEM (n = 6). Values without the same mark (a–d) represent statistically significant differences (*p* < 0.05). gg, Gallus gallus; PLCβ2, phospholipase Cβ2; IP3R3, type 3 inositol-1,4,5-trisphosphate receptor; Denatonium benzoate- Low Dose treated group, Denatonium benzoate- Medium Dose treated group, Denatonium benzoate- High dose treated group.

RT and qRT-PCRs results showed that after dietary exposure to DB the expressions of ggTas2R1, ggTas2R2 and ggTas2R7 in the hearts of chickens were significantly (*p* < 0.05) close on days 07 and 28 for all three different DB doses, with some slight subsequently higher expressions in the BD high and medium dose groups, respectively (Figure 5A,B). We observed however correlated expressions of bitter taste receptor genes, which were gradually up-regulated in a dose-dependent manner, with exception in ggTas2R7 on day 28, while the rest of the genes were similarly up-regulated (Figure 5A,B).

The expressions of downstream signaling effectors (α-gustducin, PLCβ2, IP3R3, TRPM5) in the heart of chickens on day 07 were significantly (*p* < 0.05) higher in most of the DB-treated groups than control (Figure 5A,B). whereas, the low- and high-dose DB groups on day-07 displayed significantly (*p* < 0.05) enhanced expressions of α-gustducin, PLCβ2 and IP3R3, while only the low-dose group displayed higher expressions of TRPM5 (Figure 5A,B), respectively.

The expressions of downstream signaling effectors on day-28 were only significantly (*p* < 0.05) up-regulated for α-gustducin, in the medium and high dose DB groups, while PLCβ2, IP3R3 and TRPM5 were significantly (*p* < 0.05) down-regulated in all DB-treated groups compared to control (Figure 5A,B). Nevertheless, the high DB (100 mg/kg) groups still exhibited significantly (*p* < 0.05) increased mRNA expressions of ggTas2R1, ggTas2R2, ggTas2R7, α-gustducin, PLCβ2, IP3R3, TRPM5 in contrast with the control group (*p* < 0.05; Figure 5A,B).

#### 3.4.2. Correlation Analysis

Correlation analyses (Table 2 and Table 3), exhibited a highly positive correlation among bitter taste receptors and downstream signaling gene sets separately, which may be due to their similar biological function. Moreover, a weak negative correlation among bitter taste genes-and signaling effectors-related genes was observed, which suggests that bitter receptors and signaling effectors may function frequently antagonistically.

#### 3.4.3. The mRNA Expressions of Bitter Taste and Downstream Signaling Effectors in the Kidney

**Figure 6:**
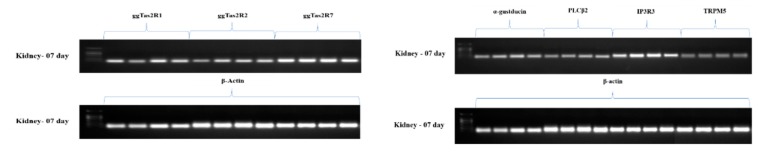

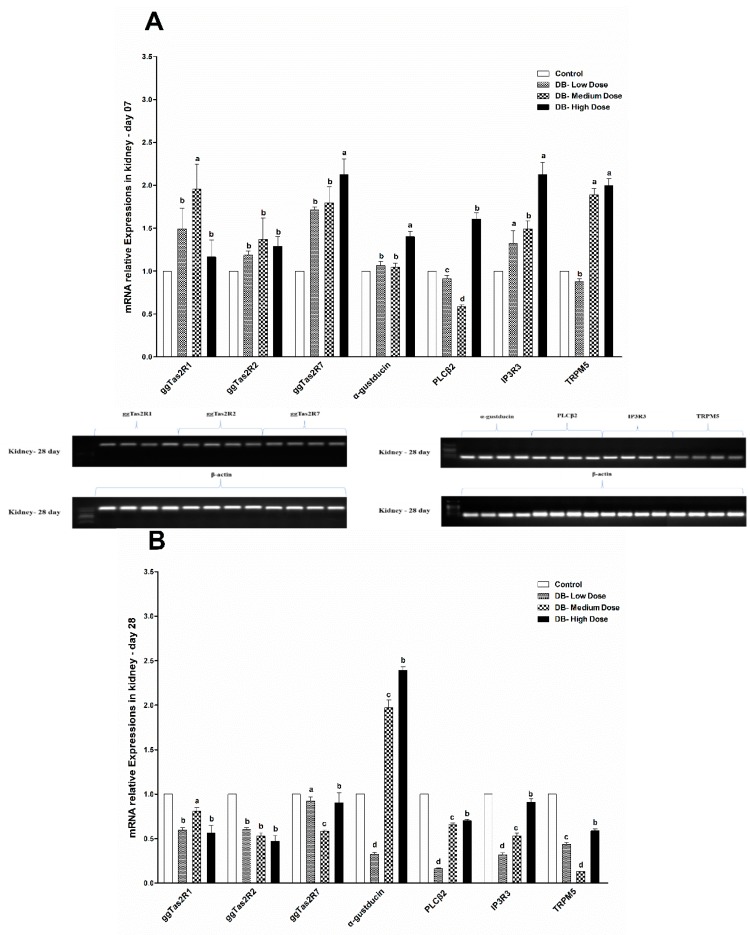
Effects of DB supplementation on kidney mRNA expressions of bitter taste receptors and downstream effectors at 07 days (**A**) and 28 days (**B**) of age. Data are presented as mean value ± SEM (n = 6). Values without the same mark (a–d) represent statistically significant differences (*p* < 0.05). gg, Gallus gallus; PLCβ2, phospholipase Cβ2; IP3R3, type 3 inositol-1,4,5-trisphosphate receptor; Denatonium benzoate- Low Dose treated group, Denatonium benzoate- Medium Dose treated group, Denatonium benzoate- High dose treated group.

ggTas2Rs showed significantly (*p* < 0.05) higher expressions in the kidney of chickens compared to the medium dose chickens, contrary to ggTas2R7, which was expressed higher in the DB high dose group on day-07 and then gradually lower expressed in day-28 with slight differences in ggTas2R7 (Figure 6A,B), respectively. 

The results indicate that ggTas2R1, ggTas2R2, ggTas2R7 have contrary expressions in kidney on day-07 and day-28 (Figure 6A,B). It was found that the expressions of ggTas2Rs were dose-dependent on DB different doses on day-07, while ggTas2R1 expressions were highly dose-dependent in the DB medium dose group among all treated groups in day-07 (Figure 6A,B). However, on day-28 the expressions of bitter taste-related genes (ggTas2Rs) were significantly (*p* < 0.05) down-regulated and there were dose-independent correlations with expressions (Figure 6B).

**Figure 7:**
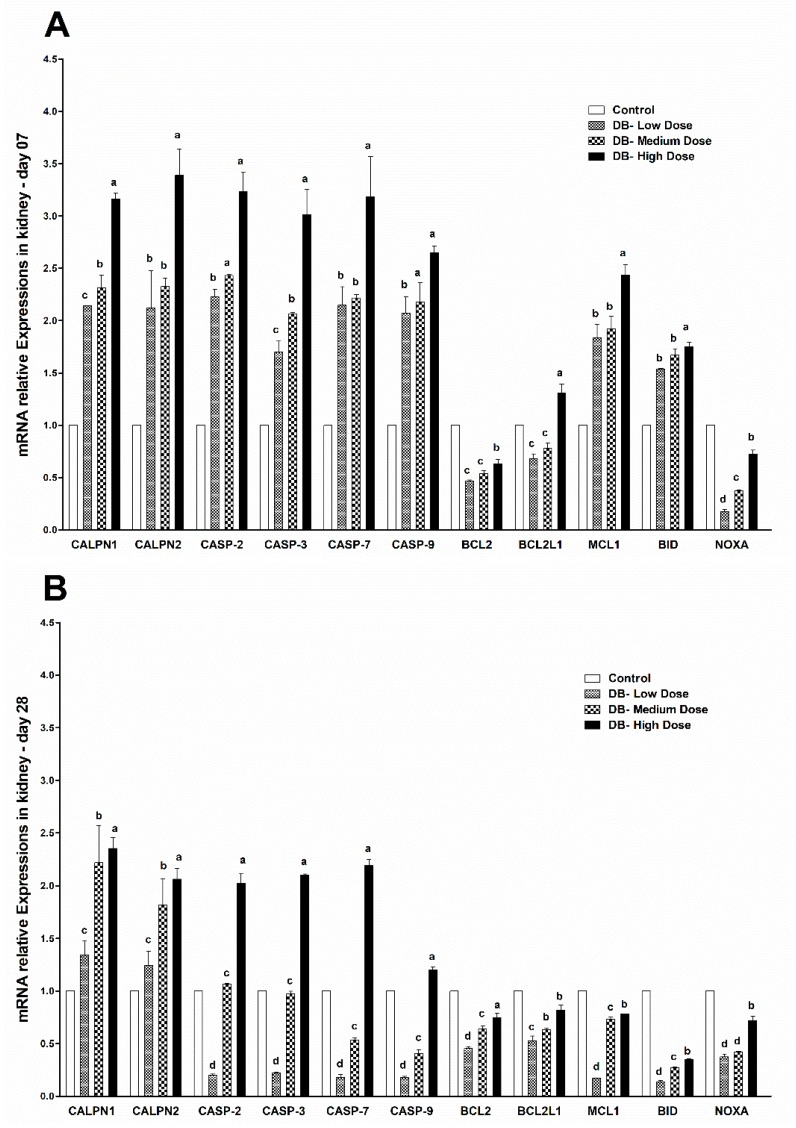
Effects of DB supplementation on kidney mRNA expressions of apoptosis-related genes at 07 days (**A**) and 28 days (**B**) of age. Data are presented as mean value ± SEM (n = 6). Values without the same mark (a–d) represent statistically significant differences (*p* < 0.05). CALPN1, Calpain 1; CALPN2, Calpain 2; CASP-2, Caspase 2; CASP-3, Caspase 3; CASP-7, Caspase 7; CASP9, Caspase 9; BCL2, B-cell CLL/lymphoma 2; BCL2L1, BCL2 like 1; MCL1, myeloid cell leukemia sequence 1; BID, BH3 interacting domain death agonist; NOXA, similar to ATL-derived PMA-responsive peptide; Denatonium benzoate- Low Dose treated group, Denatonium benzoate- Medium Dose treated group, Denatonium benzoate- High dose treated group.

On day-07 the expressions of two downstream signaling effectors genes (IP3R3, TRPM5) were significantly higher in the low, medium and high DB dose groups compared to α-gustducin and PLCβ2 (Figure 7A). However, on day-07 the high dose (100 mg/kg) DB group showed significantly higher expressions among all treated groups, exhibiting a high dose-dependent correlation (Figure 6A). In addition, on day-28 the downstream signaling effectors individually up-regulated the expression of α-gustducin in the medium and high dose DB groups, while PLCβ2, IP3R3 and TRPM5 were significantly (p < 0.05) down-regulated in all DB- treated groups compared to control (Figure 7B). Therefore, we concluded that the downstream signaling effector gene expressions were quite similar on day-07 and day-28 and no major differences were observed between them (Figure 6A,B)

### 3.5. Determination of Real Time (RT) and qRT-PCR Expressions of Apoptosis Related Genes

#### 3.5.1. The mRNA Expressions of Apoptosis Related Genes in the Heart of Chicken

**Figure 8:**
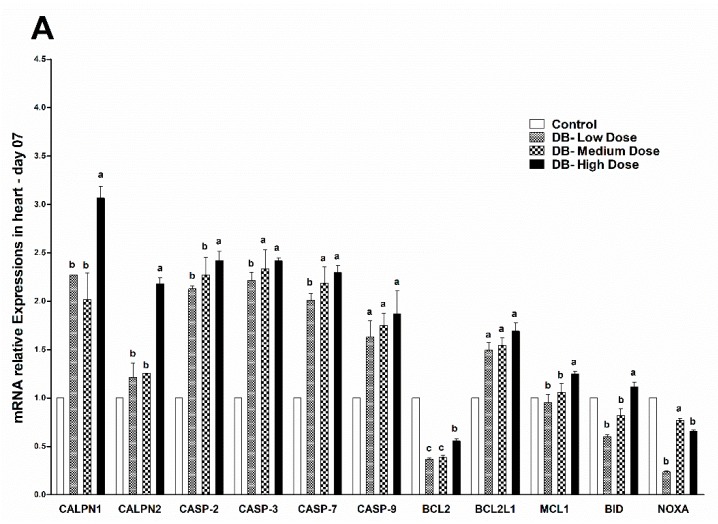

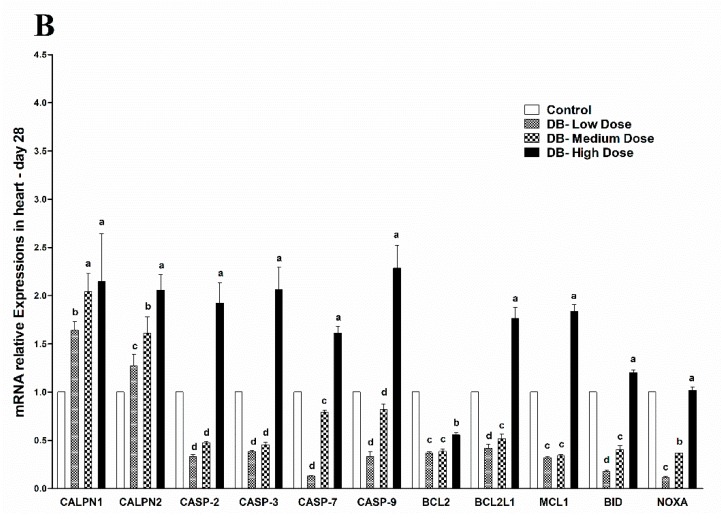
Effects of BD supplementation on heart mRNA expressions of apoptosis-related genes at 07 days (**A**) and 28 days (**B**) of age. Data are presented as mean value ± SEM (n = 6). Values without the same mark (a–d) represent statistically significant differences (*p* < 0.05). CALPN1, Calpain 1; CALPN2, Calpain 2; CASP-2, Caspase 2; CASP-3, Caspase 3; CASP-7, Caspase 7; CASP9, Caspase 9; BCL2, B-cell CLL/lymphoma 2; BCL2L1, BCL2 like 1; MCL1, myeloid cell leukemia sequence 1; BID, BH3 interacting domain death agonist; NOXA, similar to ATL-derived PMA-responsive peptide; Denatonium benzoate- Low Dose treated group, Denatonium benzoate- Medium Dose treated group, Denatonium benzoate- High dose treated group.

The qRT-PCR results showed significant (*p* < 0.05) changes in the expressions of apoptosis- related genes in both day-07 and day-28, whereas lower expressions were observed in the heart of chicken on day-28, respectively (Figure 8A,B). Comparing the CALPN1 and CALPN2 caspase family genes (CASP2, CASP3, CASP7 and CASP9) and BCL2, BCL2L1, MCL, BID and NOXA on day-07, a significantly (*p* < 0.05) higher expression pattern was observed in CALPN1 among the selected genes, that gradually reduced the CASP family-related genes and consequently the BCL2 family (Figure 8A). Lower expressions were observed in BCL2 and NOXA genes among others (Figure 8A). Therefore, the expression levels of apoptosis-related genes in day-07 were dose-dependent in the heart of chicken (Figure 8A).

On day-28 of the experiments, the apoptosis-related gene expression results revealed higher expressions of both CALPN1 and CASP9 among the genes (Figure 8B), while other genes were also significantly (*p* < 0.05) expressed, but the expressions were still lower in contrast with day-07 (Figure 8A,B). Remarkably, lower expressions on day-28 in the heart of chicken were observed in NOXA among apoptosis-related genes in the low-dose DB group (Figure 8B). To conclude, the expressions in both stages in the heart of chicken were dose- and time-dependent, hence, we found a dose-dependent relation but a time-independent relation in expressions was exhibited. Therefore, higher expressions were observed in CALPN and CASP family genes against a high dose (100 mg/kg) of DB but lower in the low-dose chickens. Thus, these genes might have similar sensitivity against DB in the heart of chicken.

#### 3.5.2. Correlation Analysis

Correlation analyses (Table 4, Table 5, Table 6 and Table 7), exhibited a highly positive correlation among apoptosis and autophagy-related gene sets in the heart and kidney of chicken, separately, which may be due to their similar biological function in selected organs. Moreover, a strong positive correlation among different apoptosis and autophagy-related genes was observed, which suggests that different apoptosis genes (CASP and BCL2 families) may function agonistically, while a very weak correlation was also observed among some genes, which suggests that they may function anti-agonistically, as shown in Table 4, Table 5, Table 6 and Table 7, respectively.

#### 3.5.3. The mRNA Expressions of Apoptosis-Related Genes in the Kidney of Chicken

After 28-days of continuous DB dietary exposure of the chickens, the results of both PCRs confirmed the expressions of apoptosis related-genes in the kidney of chickens (Figure 7A,B). On day-07, the expressions of CALPN2, CALPN1, CASP2, CASP3, CASP6, CAPS9 were significantly much higher than other apoptosis genes, respectively (Figure 8A). However, on day-07, the lowest expressions were found in NOXA among the genes in the kidney of chicken (Figure 7A). On day-28 of exposure, the highest significant expressions of apoptosis related-genes in the kidney of chicken were observed in CALPN1 and CAPS7 among the selected genes, but lower expressions were confirmed for BID, respectively (Figure 7B). The results indicate that the apoptosis-related gene expressions in the heart and kidney of chicken at the two stages of exposure were dose-dependent while we found an independent correlation with time (Figure 7A,B and Figure 8A,B).

### 3.6. Determination of Real Time (RT) and qRT-PCR Expressions of Autophagy-Related Genes

#### 3.6.1. The mRNA Expressions of Autophagy-Related Genes in the Heart of Chicken

**Figure 9:**
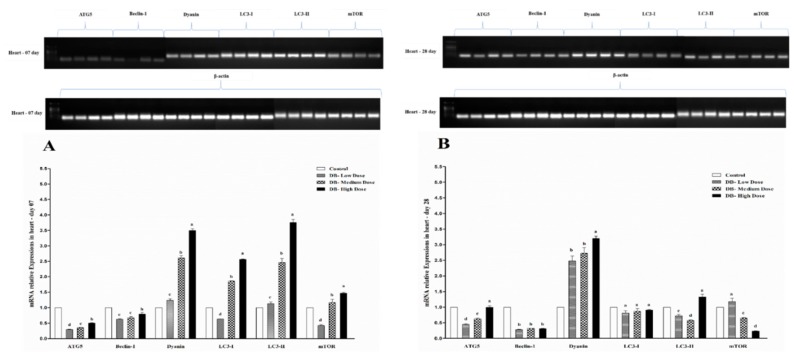
Effects of DB supplementation on heart mRNA expressions of autophagy- related genes at 07 days (**A**) and 28 days (**B**) of age. Data are presented as mean value ± SEM (n = 6). Values without the same mark (a–d) represent statistically significant differences (*p* < 0.05). ATG5; Beclin 1; Dynein; LC3-I; LC3-II, mTOR; Denatonium benzoate- Low Dose treated group, Denatonium benzoate- Medium Dose treated group, Denatonium benzoate- High dose treated group.

To determine autophagy-related genes expression in the heart of chicken, we performed RT and qRT-PCR to examine their expressions. On day-07, we observed that the lowest expressions for all DB-treated groups were for ATG5 and Beclin-1 among six selected autophagy-related genes (ATG5, Beclin-1, Dyanin, LC3-I, LC3-II, mTOR) respectively (Figure 9A,B). Meanwhile, the highest expressions of autophagy-related genes were found in LC3-II, Dyanin and LC3-I among the genes gradually (Figure 9A). Furthermore, significantly higher expressions for autophagy-related genes on day-07 was observed in the high dose (100 mg/kg) DB groups as shown in Figure 9A, whereas, the lowest expressions were exhibited in the Low-dose (5 mg/kg) DB-treated group and then in the medium-dose (20 mg/kg) DB groups (Figure 9A). We concluded from the above results that the expression levels of autophagy-related genes are directly correlated with the DB dose, therefore the expressions were dose-dependent, which lead to their higher and lower expressions in a dose-dependent manner. To evaluate the effects of DB on the autophagy-related gene expression profile in the heart of chicken we detected the expressions of six autophagy-related genes (ATG5, Beclin-1, Dyanin, LC3-I, LC3-II, mTOR) using RT and qRT-PCR techniques to confirm the expression levels. The results of both PCRs on day-28 showed that higher expressions were found in Dyanin and the lowest expression was found for Beclin-1 among all selected genes, respectively (Figure 9B). however, the expressions in ATG5, LC3-I, LC3-II and mTOR were almost similar and no major differences were observed among them (Figure 9 A,B). Notably, there was no significant differences in the expressions of ATG5, Beclin-1, LC3-I, LC3-II and mTOR and the expressions level was dose-dependent for those genes, with the only exception of mTOR, where we found a lower expression in the high dose DB group (Figure 9B). Remarkably, the expression of Dyanin was significantly higher at both stages (day-07 and day-28) in the heart of chicken among the six autophagy-related genes (Figure 9A,B). These results indicated that DB benzoate exposure aggravated ER stress and increased autophagy and the autophagic effects are dose dependent.

#### 3.6.2. The mRNA Expressions of Autophagy-Related Genes in the Kidney of Chicken

**Figure 10:**
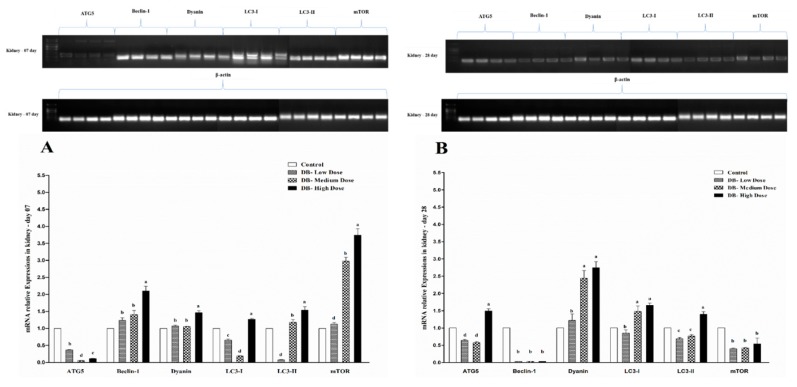
Effects of DB supplementation on kidney mRNA expressions of autophagy- related genes at 07 days (**A**) and 28 days (**B**) of age. Data are presented as mean value ± SEM (n = 6). Values without the same mark (a–d) represent statistically significant differences (*p* < 0.05). ATG5; Beclin 1; Dynein; LC3-I; LC3-II, mTOR; Denatonium benzoate- Low Dose treated group, Denatonium benzoate- Medium Dose treated group, Denatonium benzoate- High dose treated group.

The autophagy-related gene expression figures indicated that among six examined autophagy-related genes in the kidney of chicken, the expression level of mTOR gene on day-07 is dramatically increased (Figure 10A), while the rest of the genes were significantly almost equally expressed in the kidney of Chinese Fast Yellow chickens, but the higher expressions were observed in the DB high dose group for all six autophagy-related genes of the experiment with the exception of ATG5 (Figure 10A). These findings indicate that, there is direct relationship between dose and autophagy-related gene expressions in the kidney of chickens.

On day-28, higher expressions were found in Dyanin like on day-07, but lower expressions were displayed for Beclin-1 among autophagy-related genes, respectively (Figure 10B). However, the expression profile of autophagy-related genes in the kidney of chicken was dose-dependent and showed significantly gradually changing expressions (Figure 10B). Therefore, the results suggest that, autophagy-related gene expressions are dose-dependent on both day-07 and day-28 in the kidney of chicken and high dose (100 mg/kg) of DB was found to be the dose with the highest effects among the treatments.

### 3.7. Determination of Real Time (RT) and qRT-PCR Expressions of Antioxidant Genes

#### 3.7.1. The mRNA Expressions of Antioxidant-Related Genes in the Heart of Chicken

**Figure 11:**
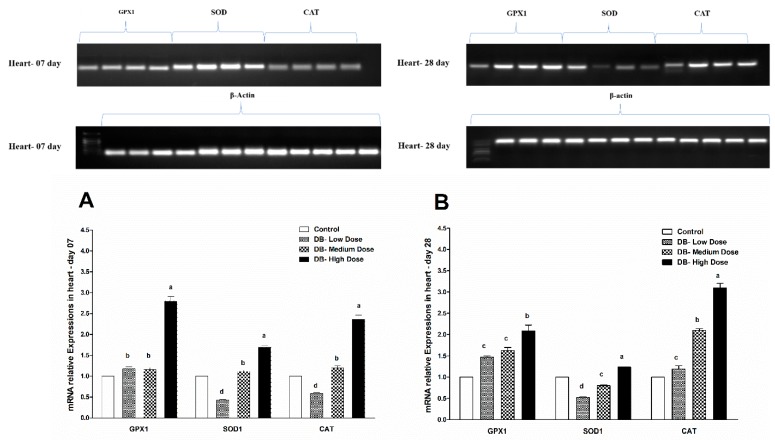
Effects of DB supplementation on heart mRNA expressions of antioxidant-related genes at 07 days (**A**) and 28 days (**B**) of age. Data are presented as mean value ± SEM (n = 6). Values without the same mark (a–d) represent statistically significant differences (*p* < 0.05). GPX1, glutathione peroxidase 1; SOD, superoxide dismutase 1; CAT, catalase; Denatonium benzoate- Low Dose treated group, Denatonium benzoate- Medium Dose treated group, Denatonium benzoate- High dose treated group.

Chicken heart is one of the organs susceptible to oxidative processes, and its oxidation state can be reflected by the levels of antioxidant gene expression, for example, glutathione peroxidase (GPx1) and catalase (CAT), which are responsible for the clearance of hydroxyl radicals [42]. Our results showed that the expression profile of oxidative stress-related genes in the heart of chicken on day-07 was significantly (*p* > 0.05) increased in a dose-dependent manner and we found high level expressions in the DB high dose (100 mg/kg) group among the three selected doses (low-dose, medium-dose and high-dose), however the expression pattern of GPX1 gene was significantly higher compared to other antioxidant genes (CAT, SOD1) (Figure 11A). On day-28, the expression level of three antioxidant-related genes were significantly (*p* > 0.05) increased consequently and higher-level expressions were detected in the DB high dose chickens, the gene expressions for GPX1, SOD1 and CAT were dose-dependent and CAT showed higher expression among them (Figure 11B). The results suggest that the heart is sensitive to oxidative stress and shows significant (*p* > 0.05) elevated expressions of antioxidative stress genes in a dose-dependent manner on both day 07 and day 28, while low-dose chickens displayed inconspicuous decreased expressions, respectively (Figure 11A,B).

#### 3.7.2. The mRNA Expressions of Antioxidant-Related Genes in the Kidney of Chicken

**Figure 12:**
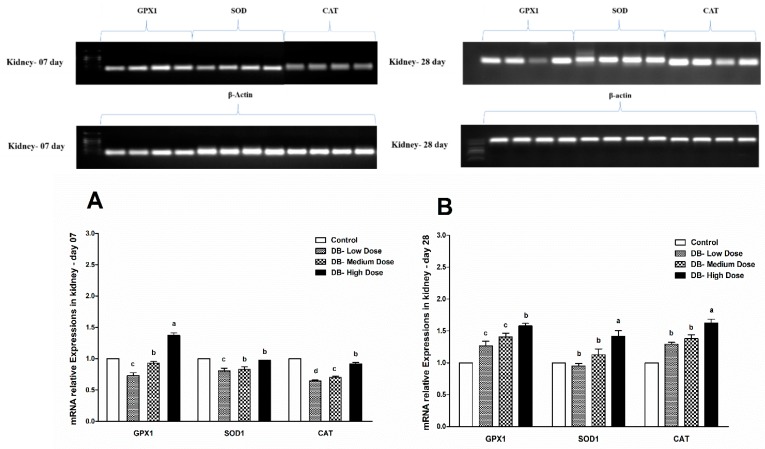
Effects of DB supplementation on kidney mRNA expressions of antioxidant- related genes at 07 days (**A**) and 28 days (**B**) of age. Data are presented as mean value ± SEM (n = 6). Values without the same mark (a–d) represent statistically significant differences (*p* < 0.05). GPX1, glutathione peroxidase 1; SOD, superoxide dismutase 1; CAT, catalase; Denatonium benzoate- Low Dose treated group, Denatonium benzoate- Medium Dose treated group, Denatonium benzoate- High dose treated group.

Because of the high sensitivity of chicken kidney to oxidative stress, the oxidation state could be reflected by the level of antioxidant genes expression profile. Most enzymatic components of this antioxidant defense system are commonly known as “antioxidant enzymes” (e.g., catalase, superoxide dismutase, glutathione peroxidase). We evaluated the expressions of such antioxidant enzymes at two different ages of chicken as a parameter to assess oxidative stress in selected organs (heart and kidney). The experimental data revealed that the expressions of three antioxidant genes (GPX1, SOD1, CAT) were expressed with approximately equally significance (*p* > 0.05) and there were slight differences in the expression level among the genes, which indicates that the expressions and sensitivity of genes related to antioxidative activity in the kidney of chicken are correlated with the dose of DB, the same as was shown in the heart of chicken, respectively (Figure 11 and Figure 12A,B).

### 3.8. Amino Acid Sequences Complete Alignment

We performed complete amino acid sequence alignment for the experiment selected genes. First, we searched for the amino acid complete sequence through exploring the NCBI database, then we did a complete alignment using two bioinformatic tools (ClustalX and Gene-Doc). The amino acid sequence alignment showed us that those genes which are identical have black color while those which are similar with each other have gray color for the alignment indications. The empty area means there is no similarity and identity among the genes, respectively. Interestingly, we found more identical and similar genes in bitter taste receptors family (ggTas2Rs), downstream signaling effectors genes and antioxidant genes, while there was little similarity among apoptosis- and autophagy- related genes. The illustrated alignment figures for bitter taste receptors genes, downstream signaling effectors genes, apoptosis related genes, autophagy related genes and antioxidant genes are shown in the Supplementary Materials.

### 3.9. Heat Map of Selected Genes

**Figure 13:**
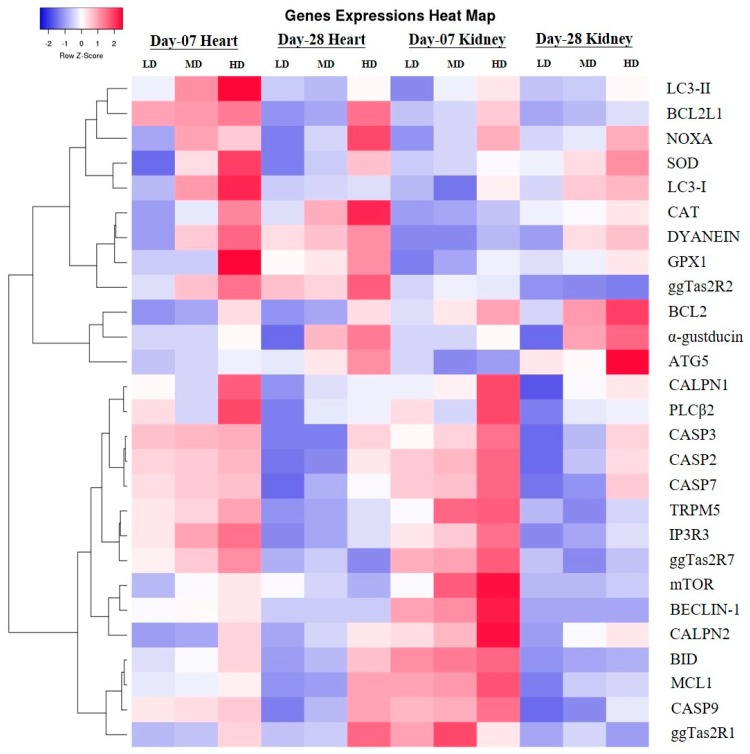
Heat map of expression profiles (bitter taste, downstream signaling effectors, apoptosis, autophagy and antioxidant genes) in supplementation with three different doses of DB in two stages (day-07 and day-28) in the heart and kidney of chicken.

We performed a heat map analysis for all selected genes of the experiment (in Figure 13). In this heat map analysis, we precisely showed all gene expressions levels and confirmed the same results as described earlier in the Results section.

## 4. Discussion

In the present study, we investigated the biological effects of denatonium benzoate (DB) on growth performance, mRNA expressions of bitter taste receptors, its downstream signaling effectors genes and related pathway, apoptosis, autophagy, antioxidant related genes, histological changes and correlations among genes expressions in the heart and kidney of Chinese Fast Yellow chickens on both day-07 and day-28 of the experiments using RT, qRT-PCR, Hematoxylin and Eosin and TUNNEL assays. We found that DB induced apoptosis, autophagy and increased the expressions of antioxidant-related genes in the heart and kidney of the chickens. However, the expressions of bitter taste receptors genes and its downstream signaling effectors were significantly higher on day-07 compared to day-28 for different DB doses, but the High-dose DB had more potential effects on apoptosis, autophagy, antioxidant, bitter taste receptors and its downstream signaling effectors gene expression than other doses, which significantly induced apoptosis and autophagy in the heart and kidney of chicken on both day-07 and day-28. Remarkably, we also found that bitter taste receptors and the associated signaling effectors, apoptosis, autophagy and antioxidant gene expressions were dependent in a dose-response manner. These findings suggest that the bitter taste receptors have a potential role among the extra-gustatory organs of the chicken, and high-dose DB causes severe necrosis via apoptosis and may result in autophagy in chicken heart and kidneys, while these symptoms were obviously observed on day-07, which proves that the chicken were more sensitive to DB exposure at the beginning of the experiments and later they adapted accordingly.

DB (485–740 mg/kg) exhibited a low toxicity rate in acute oral LD_50_ tests in rats and rabbits, while chronic toxicity studies have indicated that gavage of 16 mg/kg/day resulted in no compound-related toxicity in monkeys and rats [43,44]. In our current study, the average exposure amount of DB in the Low-dose (5 mg/kg), Medium-dose (20 mg/kg) and High-dose (100 mg/kg) groups was calculated on a daily basis for the feed and was less than the doses above. Therefore, DB dietary exposure for 28 days significantly reduced the growth performance and organ (heart and kidney) weights of the chickens. In agreement with our results, four weeks of treatment with bitter agonists like DB or quinine resulted in decreases in body weight gain associated with decreased feed intake [45]. Moreover, DB has been shown to influence ongoing interdigestive behavior, food intake and gut peptide secretion in healthy volunteers and DB may be able to suppress the contraction of smooth muscles, which inevitably affects the nutrient integration, palatability, digestibility and impairs body weight gain [33,46]. Interestingly, our results indicated that at day-07 and day-28 the ggTas2r2 expressions were higher among three tested bitter taste receptors, and separately, the higher expressions were found in the DB High-dose (100 mg/kg) group, which indicate that the expressions were dose-dependent. Overall, the expressions were significantly decreased on day-28 in contrast do day-07. This finding indicates that chicken sensitivity to DB decreased consequently.

The avian circulatory system is the main transport system of the body. It is the means by which nutrients, enzymes and other important needs for the proper functioning of body systems, organs, tissues and cells as well as body defense components are transported to where they are required. The heart is the most significant and vital organ of the avian circulatory system and its main function is blood supply/pumping of the blood [47]. To our knowledge this is the first time the potential mechanism underlying the heart pathological changes caused by different doses of DB has been determined. We performed haemotoxylin and eosin staining as well as TUNNEL assay examinations on day-07. In the haemotoxylin and eosin staining expreiments, we found particular pathological changes which alter cell necrosis, apoptosis, pyknotic cells, and distortion of morphological characteristics of the cell due to toxic effect of denatonium on the heart of chicken. Interestingly, these changes were more due to medium and high dose DB exposure. However, on day-28, we observed severe necrosis which caused apoptosis, autophagy and some shrinkage of the fibroblasts which can lead to condensation due to the effect of a high dose of BD (Figure 2A,B).

The urinary system is very complex because of its function. The kidneys maintain the water balance by removing excess water from the blood stream. Additionally, the kidneys maintain the electrolyte balance, and eliminate metabolic wastes, particularly nitrogen products. In addition, they need their own supply of nutrients for the maintenance of their own tissues and cells. When the kidneys are diseased or damaged and unable to carry out their functions efficiently, the animal becomes debilitated and death often occurs quickly [48]. The present study showed that, on day-07 and day-28, after exposure to different doses to DB the PCTs swell due to hydrophiid degeneration, the basement membrane of DCTs becomes separated and karyolysis occurred in the kidney of chickens due to the toxic effects of medium and high dose DB, respectively (Figure 3A,B). These findings were obviously more severe on day-28 compared to day-07 and suggest that after long term treatment with DB, the chicken kidney may suffer dysfunction.

Apoptosis is involved in cellular growth and development, and is important for the turnover of heart and kidney epithelial cells and tissue homeostasis [49]. Severe apoptosis is harmful for the heart and kidney, and can lead to cellular dysfunction [49,50]. It is reported that BD inhibits airway epithelial cell proliferation, decreases the number of cells and promotes cell apoptosis in a dose-dependent manner via a mitochondrial signaling pathway [19]. We performed TUNNEL assays to confirm pathological changes caused by denatonium benzoate in the heart and kidneys of chicken. As described previously, the apoptosis-related genes showed higher expressions in the heart and kidney of chicken, pathological changes that were also confirmed by haemotoxylin and eosin staining. In the present study, we detected more serious apoptosis in the heart and kidney epithelial cells of the medium and high dose DB groups. In addition, we found greater number of apoptotic cells in the high dose DB group than in the medium-dose group at 28 days, these findings revealed that denatonium benzoate amplified apoptosis in a dose-effect manner. Consistent with our results, a previous study indicated that DB inhibited airway epithelial cell proliferation, and increased cell apoptosis in a dose-effect manner [19]. Other studies also demonstrated that bitter-tasting compounds induced apoptosis in cancer cells [51]. In our study, high-dose DB exerted seriously negative effects on the heart and kidney of Chinese Fast Yellow chickens. Interestingly, low-dose DB reduce the body weight without affecting the heart and kidney epithelium after long-term adaptation. We hypothesize that low-dose DB could be added into the feed for obese layers to control the body weight due to obesity-induced dysfunctions in layers [52]. This hypothesis requires further investigations to evaluate.

The downstream signaling effectors genes (α-gustducin, PLCβ2, IP3R3 and TRPM5) of bitter taste receptors displayed similar expression patterns as the bitter taste receptors. However, these expressions were higher in day-07, while the age of chicken increased the amplified genes’ expressions (bitter and downstream signaling) in the heart and kidney of chicken were attenuated in both low-dose and medium-dose groups apparently. The results indicate that heart and kidney have a better tolerance to bitter stimuli after long-term of exposure to low and medium doses of DB. Taste transduction gene mRNA expression showed variations in the heart and kidney, through administration of DB, which suggests possible extra-gustatory effects for these genes on heart and kidney cell function of the chicken which require further investigations.

The transduction of taste is a fundamental process that allows animals to discriminate nutritious from noxious substances. Three taste modalities, bitter, sweet, and amino acid, are mediated by G protein-coupled receptors that signal through a common transduction cascade: activation of phospholipase Cβ2, leading to a breakdown of phosphatidylinositol-4,5-bisphosphate (PIP2) into diacylglycerol and inositol 1,4,5-trisphosphate, which causes release of Ca2+ from intracellular stores. The ion channel, TRPM5, is an essential component of this cascade; however, the mechanism by which it is activated is unknown. The bitter taste signaling transduction requires the involvement of Ca^2+^ influx [53,54]. It is clarified that increased cytosolic [Ca^2+^] is reversed by Ca**-ATPase [55,56]. Ca^2+^-ATPase is responsible for actively maintaining the balance of Ca^2+^ concentration within the cytoplasm and cellular organelles [57]. In the present study, the reduced activity of Ca^2+^-ATPase revealed that the function of the Ca^2+^ pump was affected in the heart and kidney. In addition, in humans and rodents, mitochondrial dysfunction and oxidative damage could cause Ca^2+^-ATPase damage [58,59]. Hence, in order to further understand the exact mechanism of Ca^2+^-ATPase damage in chicken further studies are required. Likewise, in agreement with our results in this experiment, the Ca^2+^-ATPase activity in low-dose DB and medium-dose DB groups were recovered with an adaptation to denatonium after 28 consecutive days of exposure. Moreover, excessive Ca^2+^ concentration is able to activate the Ca^2+^-dependent cysteine proteases (calpain family) [60]. The major calpain isoforms are calpain 1 and calpain 2, which are expressed in different tissues including heart and kidney of the chicken [61]. Calpain is demonstrated to be capable of inducing the activation of caspase family, which results in apoptosis [56,62]. The activation of calpain could cause tissue damage, apoptosis and autophagy [62,63]. Calpain 1 (u-calpain) and calpain 2 (m-calpain) require micromolar [Ca^2+^] and millimolar [Ca^2+^] to activate, respectively [60]. We speculate that long-term of bitter taste receptor agonist caused [Ca**]_c_ to increase in heart and kidney epithelial cells in micromolar degree according to the result of enhanced CAPN1 expression and invariant expression of CAPN2. Elevated gene expressions of CAPN1 and apoptosis executioners (BCL2, BCL2L1, Caspase 2, Caspase 3, Caspase 7, Caspase 9, MCL1, BID, NOXA) in high-dose group indicated that high-dose DB induced more apoptosis in the heart and kidney of chicken. The apoptosis result was validated by a TUNEL assays. These data increase the possibility that after administration of DB, bitter taste receptors expressed in the heart and kidney of chicken are involved in the process of apoptosis via a calpain/caspase-dependent mechanism.

Autophagy is an evolutionary conserved catabolic process that includes different forms of digestive pathways, namely macro-autophagy, micro-autophagy, chaperone-mediated autophagy and non-canonical autophagy, regulating the degradation of a cell’s own components through the lysosomal machinery [64]. Dramatically it plays a key homeostatic role in every cell type to maintain the balance between the synthesis, degradation, and consequent recycling of cellular components [65]. Currently, more than thirty different autophagy-related genes have been identified by genetic screening in yeast, and many of these genes are conserved in plants, flies and mammals, respectively [21,66]. Particularly, Bcl-2, a major apoptosis inhibitor, binds Beclin-1 to prevent its interaction with AGT5, thus resulting in the inhibition of autophagic initiation [67]. Conversely, when cleaved by caspase-3, Beclin-1 loses its ability to promote autophagy but renders cells sensitive to apoptosis [68]. Some reports indicate that autophagic degradation prevents apoptosis by eliminating harmful cellular wreckages [69,70], whereas others suggest that boosted autophagy results in increased apoptotic vulnerability [68,71]. However, data in birds are rare. Here, we report our results for the first time to indicate the autophagy-related genes expressions in the heart and kidney of chicken exposed to dietary DB treatment for 28 consecutive days. All selected autophagy-related genes in this experiment (ATG5, Beclin-1, Dyanin, LC3-I, LC3-II and mTOR) had high basal expression levels in the two examined tissues from chicken both at day-07 and day-28, respectively. However, the expressions of autophagy related genes were confirmed by RT and qRT-PCR analysis. Moreover, the expressions level of ATG5, Beclin-1 and mTOR were significantly lower in day-07 and day-28, while, we visualized higher significant expressions for Dyanin, LC3-I and LC3-II in both experimental stages in the heart of chicken. Interestingly, these expressions were in contradiction with the kidney of chicken data, where we observed higher significant expressions of Beclin-1, Dyanin and mTOR at both day-07 and day-28 among other selected genes, respectively. These results suggest that autophagy may play a crucial role in regulating many toxicity- and apoptotic-related complications which may be due to exposute to BD. On the other hand, limited knowledge is available on the role of the effect of the modulation of the autophagy process in the DB exposure context in chickens.

The endogenous cellular defense system consists of a number of antioxidant enzymes and proteins that maintain the cellular redox status, which is critical for various biological processes and functions. Most enzymatic components of this antioxidant defense system are commonly known as “antioxidant enzymes” (e.g., catalase, superoxide dismutase, glutathione peroxidase). Additionally, several experimental works evaluate the activity and expression of such antioxidant enzymes in different physiological conditions as a parameter to assess oxidative stress in a given system. As reported by Yuzhalin and Kutikhin [72]. Long-term accumulation of ROS and high levels of reactive oxygen species (ROS) may enhance oxidative damage at the DNA level. This process may affect several genes responsible for the regulation of proliferation, growth, survival, apoptosis, autophagy, invasion, leading to genomic instability and deregulation of several pathways [72]. Several enzymes, such as super oxide dismutase (SOD), glutathione peroxidase (GPX), catalase (CAT), nitric oxide synthase (NOS), and paraoxon’s (PON), function to prevent damage caused by ROS [73].

Therefore, in the present study, our results indicated different expressions level in RT and qRT-PCR analysis for the confirmation of oxidative stress in the heart and kidney of chicken due to exposure to different doses of denatonium benzoate for 28 consecutive days. However, the expressions of GPX1, SOD and CAT were almost similar on both day-07 and day-28 in the heart of chicken but we observed higher significant (*p* < 0.005) expressions among them in the BD high-dose treatment groups. This indicates that the oxidative genes expressions are dose-responsive, and it confirms our previous apoptosis and autophagy results, while similar expression patterns were observed in the kidney of chicken in day-07 and day-28 of the experiment. Remarkably, GPX1, SOD and CAT expressions were significantly (*p* < 0.005) similar, while there were slightly higher expressions in the DB high-dose treatment groups, respectively. These results suggest that oxidative stress damage is correlated with apoptotic and autophagic changes in the heart and kidney of chicken in a dose-responsive manner.

## 5. Conclusions

In summary, exposure to DB for 28 consecutive days impaired the growth performance of chickens. The present study demonstrates that dietary DB has adverse effects on the heart and kidney epithelial cells of chickens in a dose-response manner via apoptosis, autophagy and antioxidative status involving bitter taste transduction. DB can increase the oxidative stress and promote the mitochondrial apoptotic pathway via regulating ATP synthesis and mitochondrial apoptosis. Our data provide a novel perspective for understanding the interaction of heart and kidney with strong bitter taste receptor agonist. This might open a new window and maybe helpful for deeper studies of the roles and underlying mechanisms of bitter taste receptors in chickens and could have a great contribution for the improvement of chicken feedstuffs.

## Figures and Tables

**Table 1:** Feed formulation for the entire period of experiment (d 1–d 28).

Item	Diet 1 to 07 d	07 to 28 d
Ingredient (%)		
Corn	61.52	74.00
Soybean meal	29.00	12.00
Soybean oil	2.44	2.60
Corn gluten meal	2.00	7.32
Dicalcium phosphate	1.68	1.02
Premix	1.50	1.00
Limestone	1.15	1.05
Lysine sulfate	0.51	0.80
Methionine	0.20	0.21
**Total**	**100**	**100**
Calculation of nutrients		
Metabolizable energy, MJ/kg	11.92	12.13
Crude protein, %	21.00	19.00
Lysine, %	1.10	0.97
Methionine, %	0.46	0.40
Methionine + Cystine, %	0.80	0.72
Calcium, %	1.00	0.90
Available phosphorus, %	0.70	0.65
Denatonium benzoate mg/kg	5, 20, 100	5, 20, 100

The complete diet provided the following contents (% per kilogram): vitamin A, 12,500 IU; vitamin D3, 2,500 IU; vitamin E, 30 IU; vitamin K3, 2.65 mg; vitamin B_1_, 2 mg; vitamin B_2_, 6 mg; nicotinic acid, 50 mg; pantothenic acid, 12 mg; vitamin B_6_, 4 mg; folic acid, 1.25 mg; vitamin B_12_, 0.025 mg; biotin, 0.25 mg; Fe, 50 mg; Zn, 75 mg; Mn, 100 mg; Cu, 8 mg; I, 0.35 mg; Co, 0.2 mg; and Se, 0.15 mg.

**Table 2:** Correlation analysis of bitter taste and downstream signaling effectors related genes in the heart of chicken.

Gene	ggTas2R1	ggTas2R2	ggTas2R7	α-Gustducin	PLCβ2	IP3R3	TRPM5
**ggTas2R1**		0.857 *	0.717 *	0.263 *	0.169 *	−0.117 *	0.857 *
**ggTas2R2**	0.857 *		0.877 **	0.611 *	0.294 *	0.230 *	0.717 **
**ggTas2R7**	0.717 *	0.877 **		0.877 **	0.720 **	0.322 *	0.272 *
**α-gustducin**	0.263 *	0.611 *	0.877 **		0.263 *	0.611 *	0.720 **
**PLC** **β2**	0.169 *	0.294 *	0.720 *	0.263 *		0.629 *	0.681 *
**IP3R3**	−0.117 *	0.230 *	0.322 *	0.611 *	0.629 *		0.169 *
**TRPM5**	0.857 *	0.717 **	0.272 *	0.720 **	0.681 *	0.169 *	

** Correlation is highly significant at the 0.01 level (2-tailed), while * correlation is less significant.

**Table 3:** Correlation analysis of bitter taste and downstream signaling effectors related-genes in the kidney of chicken.

Gene	ggTas2R1	ggTas2R2	ggTas2R7	α-Gustducin	PLCβ2	IP3R3	TRPM5
**ggTas2R1**		0.857 *	0.717 **	0.263 *	0.169 *	−0.117 *	0.857 *
**ggTas2R2**	0.857 *		0.877 **	0.611 *	0.294 *	0.230 *	0.717 *
**ggTas2R7**	0.717 *	0.877 **		0.720 *	0.322 *	0.272 *	0.263 *
**α-gustducin**	0.263 *	0.611 *	0.720 *		0.629 *	0.681 *	0.169 *
**PLC** **β2**	0.169 *	0.294 *	0.322 *	0.629 *		0.863 *	−0.117 *
**IP3R3**	−0.117 *	0.230 *	0.272 *	0.681 *	0.863 *		0.230 *
**TRPM5**	0.857 *	0.717 **	0.263 *	0.169 *	−0.117 *	0.230 *	

** Correlation is highly significant at the 0.01 level (2-tailed), while * correlation is less significant

**Table 4:** Correlation analysis of apoptosis related-genes in the heart of chicken.

Gene	CALPN1	CALPN2	CASP2	CASP3	CASP7	CASP9	BCL2	BCL2L1	MCL1	BID	NOXA
**CALPN1**		0.972 **	0.946 **	0.350 *	0.493 *	0.783 **	0.972 **	0.893 **	0.184 *	0.360 *	0.756 **
**CALPN2**	0.972 **		0.946 **	0.893 **	0.587 *	0.693 *	0.817 **	0.184 *	0.587 *	0.895 **	0.448 *
**CASP2**	946 **	0.946 **		0.493 *	0.360 *	0.693 *	0.895 **	0.494 *	0.783 **	0.756 **	0.817 **
**CASP3**	0.350 *	0.893 **	0.493 *		0.972 **	0.946 **	0.350 *	0.493 *	0.783 **	0.972 **	0.893 **
**CASP7**	0.493 *	0.587 *	0.360 *	0.972 **		0.184 *	0.360 *	0.756 **	0.946 **	0.893 **	0.587 *
**CASP9**	0.783 **	0.693 *	0.693 *	0.946 **	0.184 *		0.693 *	0.817 **	0.350 *	0.184 *	0.587 *
**BCL2**	0.972 **	0.817 **	0.895 **	0.350 *	0.360 *	0.693 *		0.895 **	0.448 *	0.493 *	0.360 *
**BCL2L1**	0.893 **	0.184 *	0.494 *	0.493 *	0.756 **	0.817 **	0.895 **		0.693 *	0.895 **	0.494 *
**MCL1**	0.184 *	0.587 *	0.783 **	0.783 **	0.946 **	0.350 *	0.448 *	0.693 *		0.783 **	0.756 **
**BID**	0.360 *	0.895 **	0.756 **	0.972 **	0.893 **	0.184 *	0.493 *	0.895 **	0.783 **		0.817 **
**NOXA**	0.756 **	0.448 *	0.817 **	0.893 **	0.587 *	0.587 *	0.360 *	0.494 *	0.756 **	0.817 **	

** Correlation is significant at the 0.01 level (2-tailed), while * correlation is less significant.

**Table 5:** Correlation analysis of apoptosis related-genes in the kidney of chicken.

Gene	CALPN1	CALPN2	CASP2	CASP3	CASP7	CASP9	BCL2	BCL2L1	MCL1	BID	NOXA
**CALPN1**		0.972 **	0.946 **	0.350	0.493	0.783 **	0.972 **	0.893 **	0.184	0.360	0.756 **
**CALPN2**	0.972 **		0.946 **	0.893 **	0.587	0.693 *	0.817 **	0.350	0.184	0.587	0.895 **
**CASP2**	0.946 **	0.946 **		0.448	0.493	0.360	0.693 *	0.895 **	0.494	0.783 **	0.756 **
**CASP3**	0.350	0.893 **	0.448		0.817 **	0.448	0.494	0.972 **	0.946 **	0.350	0.493
**CASP7**	0.493	0.587	0.493	0.817 **		0.783 **	0.972 **	0.893 **	0.184	0.360	0.756 **
**CASP9**	0.783 **	0.693 *	0.360	0.448	0.783 **		0.946 **	0.893 **	0.587	0.693 *	0.817 **
**BCL2**	0.972 **	0.817 **	0.693 *	0.494	0.972 **	0.946 **		0.350	0.184	0.587	0.895 **
**BCL2L1**	0.893 **	0.350	0.895 **	0.972 **	0.893 **	0.893 **	0.350		0.448	0.493	0.360
**MCL1**	0.184	0.184	0.494	0.946 **	0.184	0.587	0.184	0.448		0.693 *	0.895 **
**BID**	0.360	0.587	0.783 **	0.350	0.360	0.693 *	0.587	0.493	0.693 *		0.494
**NOXA**	0.756 **	0.895 **	0.756 **	0.493	0.756 **	0.817 **	0.895 **	0.360	0.895 **	0.494	

** Correlation is highly significant at the 0.01 level (2-tailed), while * correlation is less significant

**Table 6:** Correlation analysis of autophagy-related-genes in the heart of chicken.

Gene	ATG5	Beclin-1	Dyanin	LC3-I	LC3-II	mTOR	ATG5
**ATG5**		0.972 **	0.946 **	0.350 *	0.493 *	0.783 **	0.972 **
**Beclin-1**	0.972 **		0.893 **	0.184 *	0.360 *	0.756 **	0.946 **
**Dyanin**	0.946 **	0.893 **		0.587 *	0.693 *	0.817 **	0.350 *
**LC3-I**	0.350 *	0.184 *	0.587 *		0.292 *	0.589	0.057 *
**LC3-II**	0.493 *	0.360 *	0.693 *	0.292 *		0.693 *	0.895 **
**mTOR**	0.783 **	0.756 **	0.817 **	0.589 *	0.693 *		0.494 *
**ATG5**	0.972 **	0.946 **	0.350 *	0.057 *	0.895 **	0.494 *	

** Correlation is highly significant at the 0.01 level (2-tailed), while * correlation is less significant

**Table 7:** Correlation analysis of autophagy-related-genes in the kidney of chicken.

Gene	ATG5	Beclin-1	Dyanin	LC3-I	LC3-II	mTOR	ATG5
**ATG5**		0.972 **	0.946 **	0.350 *	0.493 *	0.783 **	0.972 **
**Beclin-1**	0.972 **		0.893 **	0.184 *	0.360 *	0.756 **	0.946 **
**Dyanin**	0.946 **	0.893 **		0.587 *	0.693 *	0.817 **	0.350 *
**LC3-I**	0.350 *	0.184 *	0.587		0.895 **	0.448 *	0.493 *
**LC3-II**	0.493 *	0.360 *	0.693 *	0.895 **		0.494 *	0.783 **
**mTOR**	0.783 **	0.756 **	0.817 **	0.448 *	0.494 *		0.494 *
**ATG5**	0.972 **	0.946 **	0.350 *	0.493 *	0.783 **	0.494 *	

** Correlation is highly significant at the 0.01 level (2-tailed), while * correlation is less significant

## Supplementary Materials

The following are available at https://www.mdpi.com/2076-2615/9/9/701/s1.

## Abbreviations

DB	Denatonium Benzoate
ggTas2R1	Gallus Gallus Taste 2 Receptor 1
ggTas2R2	Gallus Gallus Taste 2 Receptor 2
ggTas2R7	Gallus Gallus Taste 2 Receptor 7
RT-PCR	Reverse Transcriptase Polymerase Chain Reaction
qRT-PCR	Quantitative Real Time; Polymerase Chain Reaction
DNA	Deoxyribonucleic Acid
RNA	Ribonucleic Acid
cDNA	Complementary Deoxyribonucleic Acid
