# Supplementary material for: Denatonium Benzoate-Induces Oxidative Stress in the Heart and Kidney of Chinese Fast Yellow Chickens by Regulating Apoptosis, Autophagy, Antioxidative Activities and Bitter Taste Receptor Gene Expressions
Source: Animals (Basel). 2019 Sep 19;9(9):701. doi: 10.3390/ani9090701 (PMC6770773; doi:10.3390/ani9090701)

Primers and 27 selected genes Amino Acid sequences complete alignment with using bioinformatics tools (ClustalX and Gene-Doc)

Table S1. Oligonucleotide PCR primers.

Gene	Accession No	Forward Primer (5 – 3)	Reverse Primer (3- 5)	Product size (bp)
ggTas2R1	AB249766.1	GGTGCCATCAAGACAGTCTTCTC	ACAGGCAGCCACTACAACAACA	135
ggTas2R2	AB249767.1	GCGATGATTCCATGGCTGC	CGTTGACCTGCAGAGGTAGG	102
ggTas2R7	NM_001080719.1	TGGCAGAGCAGCACAACACAAC	TACAAGACGCAGCCACAATGAA	111
BCL2	NM_205339.1	GAGTTCGGCGGCGTGATGTG	TTCAGGTACTCGGTCATCCAGGTG	92
BCL2L1	NM_001025304.1	TGGAGAGCGTGGACAAGGAGATG	CTCGGCAGCAGCGTTGTTCC	152
MCL1	XM_001233734.1	GTGAGCCAGTGAGCACCAACAG	ACAACCACAACTCTGAAGCATCGG	96
BID	NM_204552.2	GTCACAGGCAGTGGAAGGACTTG	GTGCTGAAGACAGGCTGTAGAAGG	138
NOXA	XM_001233389.1	CGAACCGTGCCGAGCTGTTG	TCCGCCTTGTCTCCGATCCTG	135
CAPN1	NM_001044672.1	CAGACGATCCTCAACCGCATCATC	GTTGACCATACTGCGACACGACTC	87
CAPN2	NM_205080.1	TCGGAGGAAGCAGAGGAAGATGG	CCGGAGGTTGATGAAGGTGTTGG	163
CASP2	NM_001167701.1	TCCTCCGATTCCTCCAGTGAAGC	CACAGTCCACATCTCCACCTGAAC	170
CASP 9	XM_424580.6	TGATGCTCTCCAGAGGTGTCTTCC	CCACTGAATCCTCCAATCCACTGC	195
CASP 3	NM_204725.1	TACCGGACTGTCATCTCGTTCAGG	ACTGCTTCGCTTGCTGTGATCTTC	166
CASP 7	XM_421764.5	TTCCTGGAGGAATCCTGGAAGAGG	TGAAGCGTGGATCATCAGACTGTG	158
ATG5	NM_001183963.1	AGACGCCTATTCCGTGGAATTACC	GATAGTTGGTGGCAACGAATCGC	158
Beclin-1	NM_001006332.1	ACCGCAAGATTGTGGCTGAAGAC	TGAGCATAACGCATCTGGTTCTCC	163
Dynein	NM_001006519.1	ACAATGACCTGAGGTGCCAACTG	CCACTCATCACTGGGAGGAACTG	170
LC3-I	NM_001031451.1	TTACACCCATATCAGATTCTTG	ATTCCAACCTGTCCCTCA	130
LC3-II	NM_001031461.1	AGGTGTACGAGAGCGAGAAGGAC	TACTGAGAGCGTAGGTGACATCCG	200
mTOR	XM_417614.5	AACCACTGCTCGCCACAATGC	CATAGGATCGCCACACGGATTAGC	120
PLCβ2	XM_025151351.1	GCTGTTGGTGGTGTGCCTCAG	GCTGCCTGCTTGGACTCTGTTAC	101
IP3R3	XM_418035.6	CTGGTCTGCTATTGCCGCTGTC	TTCTTGTCGCTCGTCATCAGGTTG	192
α-gustducin	NM_001267811.1	AGGAGTCAGCAAGGAGATCCAGAG	AATGATAGGTCGGAACTCCATGCG	191
TRPM5	XM 003641321.2	ATGGTGGAACAGGCAGCATTGAG	ACAGCTTCTGGCTTGACGAGTTG	190
GPX1	NM_001277853.2	AAGTGCTGCTGGTGGTCAACG	GTTGGTGGCGTTCTCCTGGTG	155
SOD1	NM_205064.1	GGTGACCTCGGCAATGTGACTG	AATGATGCAGTGTGGTCCGGTAAG	93
CAT	NM_001031215.1	ACGTTGGCGGTAGGAGTCTGG	GTGGTCAAGGCATCTGGCTTCTG	184
β-actin	NM_205518.1	CCAGCCATGTATGTAGCCATCCAG	ACGGCCAGCCAGATCCAGAC	162

∗gg–Gallus Gallus; TasR – Taste Receptor, β-actin- Housekeeping gene

27 Selected genes Amino Acid sequences complete alignment with using bioinformatics tools (ClustalX and Gene-Doc)

We performed complete amino acid sequence alignment for the experiment selected genes. First, we search amino acid complete sequence through exploring NCBI database, then we do complete alignment using two bioinformatics tools (ClustalX and Gene-Doc). The amino acid sequence alignment shows us that, those genes which are identical have black color while those which are similar with each other have gray color for the alignment indications. The empty area means there is no similarity and identity among the genes respectively. Interestingly, we found more identical and similar genes in bitter taste receptors family (ggTas2Rs), downstream signaling effectors genes and antioxidant genes, while there was rare similarity among apoptosis and autophagy related genes. The illustrated alignment figures for bitter taste receptors genes, downstream signaling effectors genes, apoptosis related genes, autophagy related genes and antioxidant genes are shown below.

1. Bitter Taste Receptors genes complete sequence alignment (ggTas2R1, ggTas2R2 and ggTas2R7)

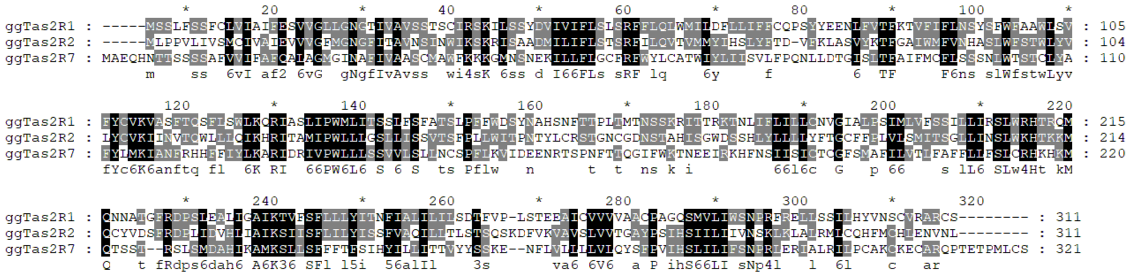

2. Apoptosis related genes sequences complete alignment

```
                  *        20         *        40         *        60         *        80         *       100         *
BCL2   : -------------------------------------------------------MAHPGRRGYDNREIVLKYIHYKLSQR----------------------------- :  26
BCL2L1 : -------------------------------------------------------MSSS------NRELVIDFVSYKLSQR----------------------------- :  20
CALPN1 : MAEEPVVPVFCTGVSAQVQRQRAKALGLGQHQNAVRFRGQDYAALRDDCLRSGSLFRDETFPPSASSLGFRELGPGSSKTR----------------------------- :  81
CALPN2 : ----------MAGMAAALAKERAAAAGAGRHGQAVPYLGQDFGALRRECLQGGRLFHDPSFPAGPAALGYRELGPNSYKTK----------------------------- :  71
CASP3  : -------------------------------------------------------MMTDIKDGPRSGE-------DVSDAR----------------------------- :  19
CASP7  : -------------------------------------------------------MSGDQHADRSSGEKSNGDQDDTVDAK----------------------------- :  26
CASP9  : -----------------MEEAQRSALRRGRVRLVEGLRPEVLWGPLQARGVFTSAMVEDMQSTGTRREQARQLVIDLETRGKQAFPIFLSILRDTGHGDLADMLDEGCGS :  93
CASP2  : -----------MLGACGMQRYHQEALKKNRVMLARELVLKELMEHMIEKDIITIEMVEMIQAKSGSFSQNVEFLNLLPKRGPNAFSAFCEALQETKQQHLAEMILKTESS :  99
MCL1   : -------------------------------------------------------------------------------------------------------------- :   -
NOXA   : -------------------------------------------------------------------------------------------------------------- :   -
BID    : -------------------------------------------------------------------------------------------------------------- :   -

                120         *       140         *       160         *       180         *       200         *       220
BCL2   : ----------------------GYDWAAGEDRPPVPPAPAPAAAPAAVAAAGA----SSHHRPSPPARLLLVR--CPRLRGCAAPPG--VHLALRQAGDEFSRRYQRDFA : 106
BCL2L1 : ----------------------GHCWSELEEEDENRTDTAAEAEMDSVLNGSP----SWHPPAGHVVNGATVHRSSLEVHEIVRASD--VRQALRDAGDEFELRYRRAFS : 102
CALPN1 : ----------------------GVTWKRPTELCRHPQFIVDGATRTDICQGALGDCWLLAAIASLTLNETILHRVVPHGQSFQNGYAGIFHFQIWQFGEWQDVVVDDYLP : 169
CALPN2 : ----------------------GVVWCRPTELCSCPRFIAGGATRTDICQGALGDCWLLAAIASLTLNEEILARVVPRDQSFQDEYAGIFHFQFWQYGEWVDVVVDDRLP : 159
CASP3  : ------------------------SFPGSKGMNLPASKSVDSG--------------ILPDDSYRMDYPEIGVCVIINNKNFHRDTG----LSSRSGTDADAASVREVFM :  87
CASP7  : ------------------------PDRSSR-LSLFAKKKKNGEEEQPKSSLSNQYRIVTPTFQYNMNYKKVGKCIIINNKNFEDKTG----MGTRNGTDKDAGDLSKSFR : 107
CASP9  : PMS--PPVDLRPVQLELPGDRRDKSVSTAERLSIPVQPESERFRMPPAPAQGSAVDKSDHRLVYQLRADPCGHCLIFNNVSFSRDSD----LSTRAGSDIDCEKLEKRFR : 197
CASP2  : LRHGIATLEQRYGSNLPLPLESCNSKRPRLIVEHSLDSGDGPPIPPVKHCTPEFYRDHQHLAYKLISEPRGLALILSNIHFSSEKD----LEYRSGGDVDCASLELLFK : 205
MCL1   : -------------------------------------------------------------------------------------------------------------- :   -
NOXA   : -------------------------------------------------------------------------------------------------------------- :   -
BID    : ---------------------------------------------MEQDIYSNGSDHMERMLLFAFLAESSGCEFKEQLPSLQSQGVLCSVKDALCYDSDGELQTDGNRS :  65

                  *       240         *       260         *       280         *       300         *       320         *
BCL2   : QMSGQLHLTPFTATGRFVAVVEELFRDGVN--------WVRIVAFFEFGG-------------------------------VMCVESVNREMSPLVDNIATWMTEYLN-- : 175
BCL2L1 : DLTSQLHITPGTAYQSFEQVVNELFHDGVN--------WGRIVAFFSFGG-------------------------------ALCVESVDKEMRVLVGRIVSWMTTYLT-- : 171
CALPN1 : TKDGKLLFVHSAEGTEFWSALLEKAYAKVNGCYEALSGGSTSEGFEDFTGGVTEWYDLRKPPADLYQIILKALERGSLLGCSIDITSAFDMEAVTFKKLVKGHAYSVTGA : 279
CALPN2 : TKNGELLFVHSAEGSEFWSALLEKAYAKLNGSYEALSGGTTTEGFEDFTGGIAEWYELQKAPPNLFKIIQKALQKGSLLGCSIDITSAAETEAVTSQKLVKGHAYSVTGA : 269
CASP3  : KLGYKVKLNNDLSSRDIFKLLKNVSEEDHS------KRSSFVCVLLSHGDE----------------------------GLFYGTDG-PLELKVLTSLFRGDKCRSLA-- : 160
CASP7  : SLGFDVYTYNDRSCEDMQTLLKQAAEENHS------DAACFACILLSHGEE----------------------------GLIYGTDG-PMAIKHLTTLFRGDKCKTLI-- : 180
CASP9  : SLCFHVRTLRNLKAQEIDVELRKLARLDHS------ALDCCLVVILSHGCQTSHI---------------------QFPGGIYGTDGKIIPIERIVNYFNGSQCPSLR-- : 278
CASP2  : HLGYQVTVFHDQSAEEMESALERFSKLPDH-----QDVDSCIVALLSHGVEG----------------------------GVYGTDGKLLQLQEAFRLFDNANCPNLQ-- : 280
MCL1   : -MLRKLEIKKEDDLQAVCEVAAHVFNDGVTN-------WGRVVTLISFGA-------------------------------FVAKHLKSINQEKCITSLAGIITDALVS- :  70
NOXA   : -------MMPGRTIRKPAPPAAPAERDAVA---------ECALELRRIGD-------------------------------------KADLQQKVLNLITKLFCPKT--- :  54
BID    : GHLQNGELVPDPEVNEAIVRTIAAQLAEIGDQLDKQIKAKVVNDLVQH---------------------------------FLNENLPREEITRCLSQAVEGLARAIP-- : 140

                340         *       360         *       380         *       400         *       420         *       440
BCL2   : ----------------------RHLHNWIQDNGGWDAFVELYGNSMRPLFDFS---------------------WISL-KTILSLVLVGACITLGAYLGHK--------- : 232
BCL2L1 : ----------------------DHLDPWIQENGGWERFVDLYGNNAAAELRKG---------------------QETFNKWLLTGATVAGVLLLGSLLSRK--------- : 229
CALPN1 : KQISYRGQSLGLIRMRNPWGEVEWTGAWSDNSSEWNAVEPALRQQLMVRMEDGE-------------------FWMSFRDFLREFTRIEICNLTPDALQSRKFRKWNTRL : 370
CALPN2 : EEVNFRGSIQKLIRIRNPWGEVEWTGKWNDNCPNWSGVDPEVRERLTRRHEDGE-------------------FWMAFNDFLRHYSRIEICNLTPDTLASDRYKKWSLLK : 360
CASP3  : ----------------------GKPKLFFIQACRGTELDSGIEADSGP-------------------------DETVCQKIPVEADFLYAYSTAPGYYSWRNA------- : 216
CASP7  : ----------------------GKPKLFFIQACRGSEFDEGIQTDSGPAND------------------TLETDANPRYKIPVEADFLFAYSTVPGYYSWRNP------- : 243
CASP9  : ----------------------GKPKLFFIQACGGEQKDQGFEVDCESPQDETCRRSIESDAIPFQAPSGNEDEPDAVASLPTPGDILVSYSTFPGFVSWRDK------- : 359
CASP2  : ----------------------NKPKMFFIQACRGDETDRGVDQRDGKEWSDSPGCE----------ESDANKEENLKLRLPTRSDMICGYACLKGTAAMRNT------- : 351
MCL1   : -----------------------SKREWLMSQGGWEGFVDFFRVEDLES---------------------------SIRNVLMAFAGVAGLGASLAYMIRKWRS------ : 124
NOXA   : -------------------------------------------------------------------------------------------------------------- :   -
BID    : --------------------------SDLEQEKAMLVLAMLLTKKVANQMP-------------------------SLLQRVFSTTVNYISQHFHNYIVRMLRE------ : 193

                  *       460         *       480         *       500         *       520         *       540         *
BCL2   : -------------------------------------------------------------------------------------------------------------- :   -
BCL2L1 : -------------------------------------------------------------------------------------------------------------- :   -
CALPN1 : YDGSWRRGSTAGGCRNYPATFWINPQFKICLEEVDDDGDDFGGREPGCSFLLALMQKHRRRERRYGKDMETIGFAVYEVPPEHVGHSGVHLQRDFFLSNASRARSEQFIN : 480
CALPN2 : LDGNWRRGATAGGCRNYPNTFWTNPQYLIKLEEEDEDPDDPEG---GCTFLIGLIQKHRRKQRKMGEDMHTIGFAIYEVPPEFSGQTNIHLSKNFFLTNKAREKSNTFIN : 467
CASP3  : AEGSWFIQSLCRMLKEHAR-------------------------------KLELMQILTRVNRRVA-EYESCSTRQDFNAKKQIPCIVSMLTKEFYFPC----------- : 283
CASP7  : GRGSWFVQSLCSVLNEHGK-------------------------------QLEIMQILTRVNYVVATNFESQSDDPRFSEKKQIPCVVSMLTKELYF------------- : 309
CASP9  : VSGSWYVETLDSVLEHYAR-------------------------------SEDLLTMLLRVSDIVS----------TKGRYKQIPGCFNFLRKKFFFLCK---------- : 418
CASP2  : KRGSWYIEALTTVFAEDSR-------------------------------DTHVADMLVKVNRQIK-QREGYAPGTEFHRCKEMSEYCSTLCRDLYLFPGYVPGK----- : 424
MCL1   : -------------------------------------------------------------------------------------------------------------- :   -
NOXA   : -------------------------------------------------------------------------------------------------------------- :   -
BID    : -------------------------------------------------------------------------------------------------------------- :   -

                560         *       580         *       600         *       620         *       640         *       660
BCL2   : -------------------------------------------------------------------------------------------------------------- :   -
BCL2L1 : -------------------------------------------------------------------------------------------------------------- :   -
CALPN1 : LREVSTRLRLPPGEYIVVPSTFEPNREGDFVLRVFSEKRADTEEIDDKIEAKLPDEKVLSEGEIDENFKQLFRQLAGPDMEISVTELQTILNRIIAKHKDLRTKGFSLES : 590
CALPN2 : LREVLNRFKLPAGEYIIVPSTFEPNLNGDFCLRVFSEKNANSTVIDDEIEANF-EETEIDEDDIEPSFKKLFGQLAGSDAEISAFELRSILNKILAKRQDIKSDGFSIET : 576
CASP3  : -------------------------------------------------------------------------------------------------------------- :   -
CASP7  : -------------------------------------------------------------------------------------------------------------- :   -
CASP9  : -------------------------------------------------------------------------------------------------------------- :   -
CASP2  : -------------------------------------------------------------------------------------------------------------- :   -
MCL1   : -------------------------------------------------------------------------------------------------------------- :   -
NOXA   : -------------------------------------------------------------------------------------------------------------- :   -
BID    : -------------------------------------------------------------------------------------------------------------- :   -

                  *       680         *       700         *       720         *       740         *       760         *
BCL2   : -------------------------------------------------------------------------------------------------------------- :   -
BCL2L1 : -------------------------------------------------------------------------------------------------------------- :   -
CALPN1 : CRSMVNLMDKDGNGKLGLVEFNVLWNRIRNYLSVFRKFDLDKSGSMSAYEMRMALEASGYKLTQKLHQLLITRYAEPDLAIDFDSFVCCLVRLETMFRFFQAMDGDNDGV : 700
CALPN2 : CKIMVDLLDNDGSGKLGLKEFHTLWTKIQKYQKIYREIDVDRSGTMNSYEMRRALEAAGFKLSCQLHQIIVARFADEDLIIDFDNCVRCLIRLETLYKMFRKLDTEKTGT : 686
CASP3  : -------------------------------------------------------------------------------------------------------------- :   -
CASP7  : -------------------------------------------------------------------------------------------------------------- :   -
CASP9  : -------------------------------------------------------------------------------------------------------------- :   -
CASP2  : -------------------------------------------------------------------------------------------------------------- :   -
MCL1   : -------------------------------------------------------------------------------------------------------------- :   -
NOXA   : -------------------------------------------------------------------------------------------------------------- :   -
BID    : -------------------------------------------------------------------------------------------------------------- :   -

                780
BCL2   : --------------- :   -
BCL2L1 : --------------- :   -
CALPN1 : VTFGLLQWLQLTMFA : 715
CALPN2 : IELNLINWLFFTVI- : 700
CASP3  : --------------- :   -
CASP7  : --------------- :   -
CASP9  : --------------- :   -
CASP2  : --------------- :   -
MCL1   : --------------- :   -
NOXA   : --------------- :   -
BID    : --------------- :   -
```

3. Autophagy related genes complete sequences alignment

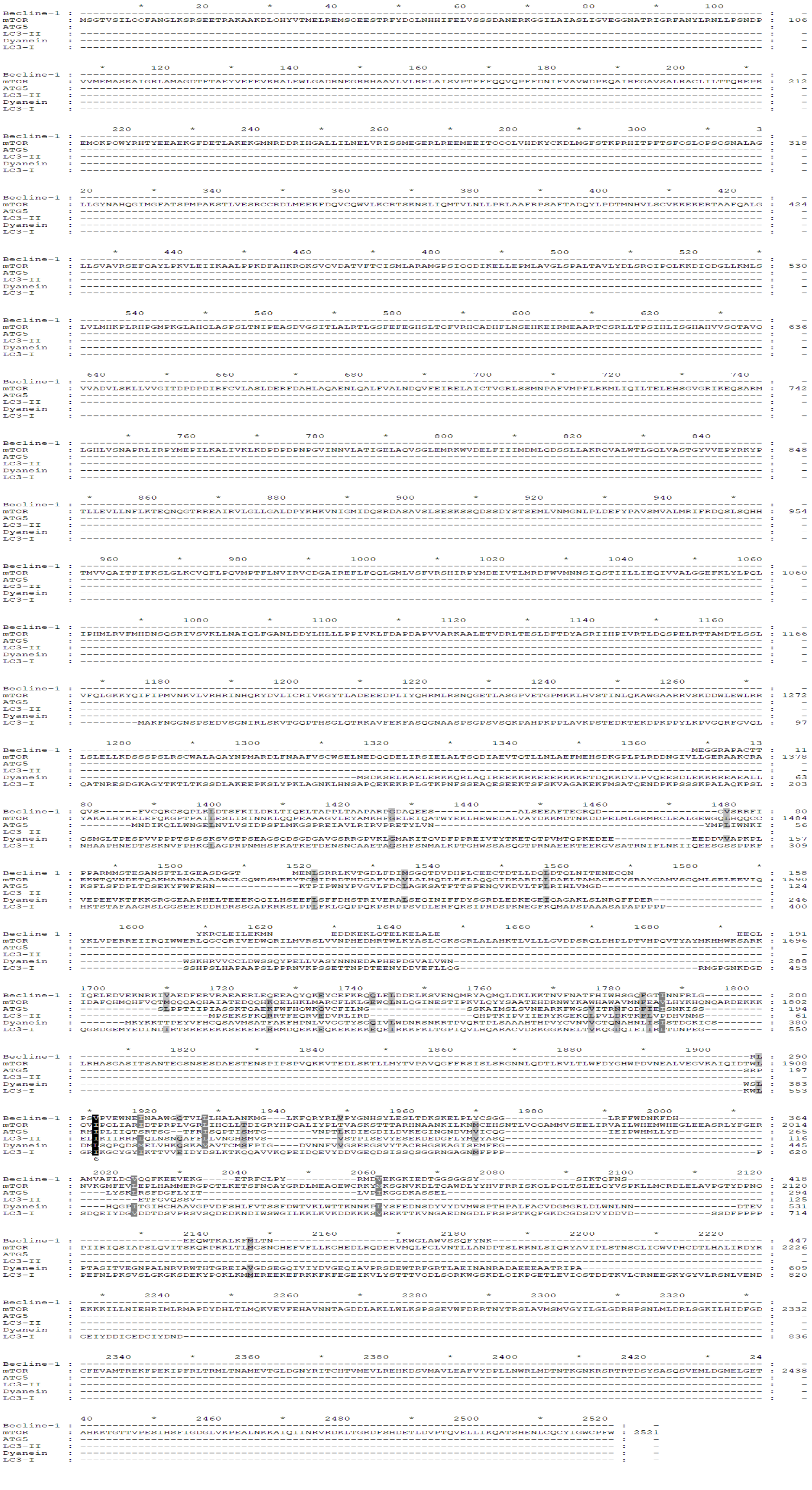

4. Antioxidant related gens sequences complete alignment

```
                *         20         *        40         *        60         *        80         *       100         *
GPX1 : -----MAATGLAGILARPLGAAEPLALSSLRG----KVLLVVNVASLUGTTTRDFLQLNELQQR---------YGPRGLRVLG-----------FPCNQFGHQENATNEEILR- :  84
CAT  : MADGRDVASEQLKRWQSQRGSQKPDALTTGAGNPIGDKLNIMTVGPRGPLLVQDVVFTDEMAHFDRERIPERVVHAKGAGAFGYFEVTHDITKYCKAKVFEHIGKRTPIAIRFS : 114
SOD  : --------------MATLKAVCVMKGDAPVEG-----VIHFQQQGSGPVKVTGKITGLSDGDHG------------------------------FHVHEFGDNTNGCTSAGAH- :  64
              a       a   g   p al    G     v6    vgs     t d   l e  h              g    g           f    Fgh  n t  ai

          120         *       140         *       160         *       180         *       200         *       220
GPX1 : -------SLEYVRPGNGFKPNFTMFEKCEVNGKGAHPLFAFLREALPFP------HDDPSALMTNPQYIIWSPVCRNDVSWNFEKFLVGPDGVP--FRRYSRH-FETIKLQDDI : 182
CAT  : TVAGESGSADTVRDPRGFAMKFYT-EEGNWDLVGNNTPIFFIRDAMLFPSFIHSQKRNPQTHLKDPD-MVWDFLSLRPESLHQVSFLYSDRGIPDGFRHMNGYGSHTFKLVNAS : 226
SOD  : ------FNPEGKQHGGPKDADRHVGDLGNVTAKGGVAEVEIEDSVISLT---------------GPHCIIGRTMVVHAKSDDLGRGGDNESKLTG---------NAGPRLACGV : 148
              s e vr g gf   f   e gnv  kG      f r a6 fp         p      P  66w  6     S     fl    g6p  fr        t 4L

        *       240
GPX1 : ELLLQKVPKEALQ : 195
CAT  : GGAVTASPR---- : 235
SOD  : IGIAKC------- : 154
        g     p
```

5. Downstream signaling effectors gene complete sequences alignment

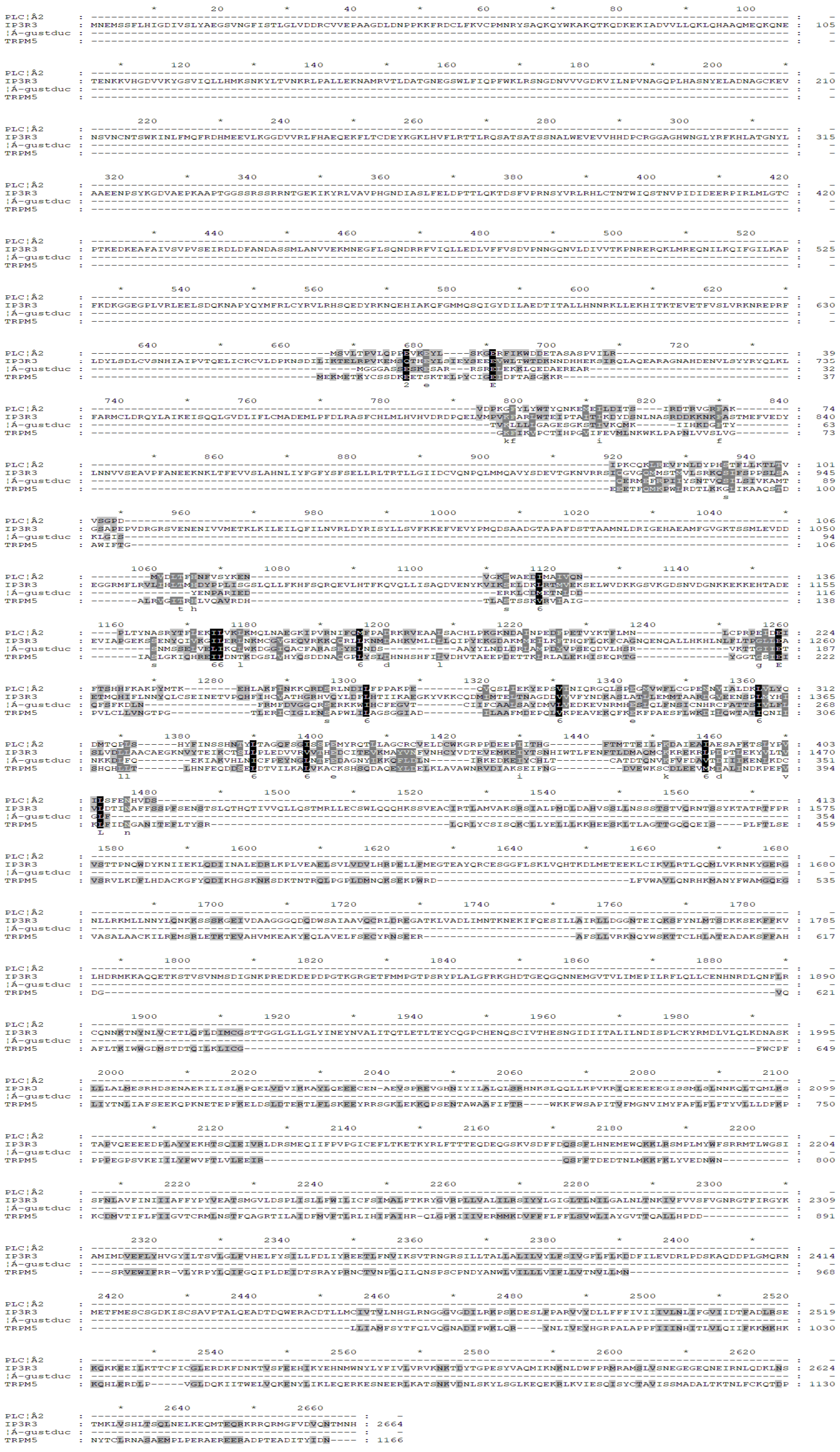

Supplement: Supplementary file 1 [file animals-09-00701-s001.pdf]
